# Atrial natriuretic factor receptor guanylate cyclase, ANF-RGC, transduces two independent signals, ANF and Ca^2+^

**DOI:** 10.3389/fnmol.2014.00017

**Published:** 2014-03-17

**Authors:** Teresa Duda, Alexandre Pertzev, Rameshwar K. Sharma

**Affiliations:** The Unit of Regulatory and Molecular Biology, Research Divisions of Biochemistry and Molecular Biology, Salus UniversityElkins Park, PA, USA

**Keywords:** atrial natriuretic factor, atrial natriuretic factor receptor guanylate cyclase, calcium, cyclic GMP, neurocalcin δ, signal transduction

## Abstract

Atrial natriuretic factor receptor guanylate cyclase (ANF-RGC), was the first discovered member of the mammalian membrane guanylate cyclase family. The hallmark feature of the family is that a single protein contains both the site for recognition of the regulatory signal and the ability to transduce it into the production of the second messenger, cyclic GMP. For over two decades, the family has been classified into two subfamilies, the hormone receptor subfamily with ANF-RGC being its paramount member, and the Ca^2+^ modulated subfamily, which includes the rod outer segment guanylate cyclases, ROS-GC1 and 2, and the olfactory neuroepithelial guanylate cyclase. ANF-RGC is the receptor and the signal transducer of the most hypotensive hormones, ANF– and B-type natriuretic peptide (BNP). After binding these hormones at the extracellular domain it, at its intracellular domain, signals activation of the C-terminal catalytic module and accelerates the production of cyclic GMP. Cyclic GMP then serves the second messenger role in biological responses of ANF and BNP such as natriuresis, diuresis, vasorelaxation, and anti-proliferation. Very recently another *modus operandus* for ANF-RGC was revealed. Its crux is that ANF-RGC activity is also regulated by Ca^2+^. The Ca^2+^ sensor neurocalcin d mediates this signaling mechanism. Strikingly, the Ca^2+^ and ANF signaling mechanisms employ separate structural motifs of ANF-RGC in modulating its core catalytic domain in accelerating the production of cyclic GMP. In this review the biochemistry and physiology of these mechanisms with emphasis on cardiovascular regulation will be discussed.

## INTRODUCTION

In the 1980s, the field of cellular signal transduction underwent total metamorphosis. Until then only two major paradigms of signal transduction, that of cyclic AMP and that of IP_3_ (inositol triphosphate) were known and were used to explain cellular signaling mechanisms and the second messenger concepts. Each of these two paradigms constituted an assemblage of three components – receptor, GTP binding protein, and the transducer catalyst – necessary for signal transduction. The astonishing discovery of a novel membrane protein that was simultaneously a receptor of a hormone and the transducer of its signal added a new dimension to our understanding of cellular signal transduction. The hormone was the newly described atrial natriuretic factor (ANF; de Bold, 1985) and the membrane protein was both its receptor and guanylate cyclase enzyme, termed therefore atrial natriuretic factor receptor guanylate cyclase (ANF-RGC; Kuno et al., 1986; Paul, 1986; Paul et al., 1987; Takayanagi et al., 1987; Meloche et al., 1988). Other terms to describe the protein are GC-A and NPR-A. ANF-RGC responded to ANF binding with accelerated synthesis of its second messenger cyclic GMP. The novelty of the system was 2-fold; first, a single protein, ANF-RGC, which contained both the ability to recognize the ANF hormonal signal and the activity to translate the hormonal information into the production of its second messenger, cyclic GMP was identified; and second, cyclic GMP was recognized as *bona fide* hormonal second messenger. Until then, the majority of laboratories forcefully denied the hormonal second messenger role of cyclic GMP (reviewed in Sharma, 2002, 2010).

The concept that ANF-RGC is indeed both hormone receptor and a catalyst was further supported by cloning studies (Chinkers et al., 1989; Lowe et al., 1989; Pandey and Singh, 1990; Duda et al., 1991; Marala et al., 1992). Homology cloning made possible finding other receptor membrane guanylate cyclases: C-type natriuretic peptide receptor guanylate cyclase, CNP-RGC (also known as GC-B; Chang et al., 1989; Schulz et al., 1989; Duda et al., 1993b) and heat-stable enterotoxin (also guanylin and uroguanylin) receptor guanylate cyclase, STa-RGC (GC-C; Schulz et al., 1990; de Sauvage et al., 1991; Singh et al., 1991; Hamra et al., 1993; Khare et al., 1994). Identification of these three structurally and functionally related membrane guanylate cyclases that were receptors for hormonally active peptides resulted in a notion that all membrane guanylate cyclases, even those still to be discovered, were receptors for specific extracellular ligands.

The notion was short-lived, however. The next four, in chronological order, identified membrane guanylate cyclases: the rod outer segment guanylate cyclases, ROS-GC1 (also known as RetGC1 or GC-E; Koch, 1991; Shyjan et al., 1992; Goraczniak et al., 1994) and ROS-GC2 (RetGC2 or GC-F; Lowe et al., 1995; Yang et al., 1995; Goraczniak et al., 1998), the olfactory neuroepithelium guanylate cyclase, ONE-GC (also termed as GC-D) (Fulle et al., 1995; Duda et al., 2001a) and GC-G (Schulz et al., 1998), did not respond with increased activity to any extracellular ligand. They were therefore branded as “orphan receptors” (Fulle et al., 1995; Yang et al., 1995; Wedel and Garbers, 1997; Schulz et al., 1998).

ROS-GC1 and ROS-GC2 were not orphan RGC, however. Biochemical and physiological findings began to reveal that regulation of their catalytic activities is specific to their physiological function which is to return the illuminated photoreceptors to the dark, resting state. The illumination leads to activation of cyclic GMP phosphodiesterase, depletion of cyclic GMP, closure of the cyclic GMP gated (CNG) channels, and lowering the free Ca^2+^ concentration (reviewed in Pugh et al., 1997; Koch et al., 2010). The ROS-GCs’ task is to restore the dark-level of cyclic GMP allowing opening of the CNG channels and increase of Ca^2+^ influx. Ca^2+^ concentration, thus, determines the activity of ROS-GCs but it occurs in an indirect way. Guanylate cyclase activating proteins (GCAPs) sense the post-illumination fall in Ca^2+^ and stimulate ROS-GCs to resynthesize cyclic GMP at a faster rate (reviewed in Detwiler, 2000; Koch et al., 2010). In this cyclic GMP-Ca^2+^ feedback mechanism, ROS-GCs do not respond to an extracellular ligand but to their intracellular Ca^2+^ sensing ligands, the GCAPs (Palczewski et al., 1994; Dizhoor et al., 1995; Duda et al., 1996b).

Soon thereafter the evidence began to emerge, primarily from our laboratory, that GCAPs are not the only Ca^2+^ sensing modulators of ROS-GC activity. While increasing Ca^2+^ concentrations inhibit ROS-GCs activity through GCAPs, two other Ca^2+^ sensors, S100B, and neurocalcin δ (NCδ) stimulate ROS-GC1 in a Ca^2+^-dependent fashion (Pozdnyakov et al., 1995; Duda et al., 1996a; Margulis et al., 1996; Kumar et al., 1999). The Ca^2+^-dependent S100B-mediated activation of ROS-GC operates in cones including their outer segments and pedicles (Wen et al., 2012). Its role in photo- and visual transductions remains to be established, but existing data indicate its involvement in transmission of the visual signal from cone ON-bipolar cells (Wen et al., 2012). Ca^2+^ signaling of ROS-GC activity mediated by NCδ is operative in retinal ganglion cells (Krishnan et al., 2004) but its linkage with the visual transduction is not clear yet.

Similar, but with additional twists, regulation of ONE-GC occurs in a subset of olfactory sensory neurons (Fulle et al., 1995; Duda et al., 2001a; Pertzev et al., 2010). The cyclase was initially classified as a member of the Ca^2+^ modulated subfamily (Duda et al., 2001a). Its activity is modulated in a Ca^2+^-dependent fashion by NCδ (Duda et al., 2001a, 2004) and by GCAP1 (Duda et al., 2006, 2012a; Pertzev et al., 2010). Remarkably, the Ca^2+^-GCAP1 pattern of ONE-GC modulation is opposite to that of ROS-GC modulation. Sensing increasing concentrations of Ca^2+^ GCAP1 stimulates ONE-GC while it inhibits ROS-GC (Duda et al., 2006, 2012a).

After the Ca^2+^-dependent modulation of ONE-GC activity was demonstrated, an extracellular ligand of the cyclase was found. The ligand was the odorant uroguanylin (Leinders-Zufall et al., 2007; Duda and Sharma, 2008; reviewed in Sharma and Duda, 2010; Zufall and Munger, 2010). Hence, ONE-GC responds to both extracellular (uroguanylin) and intracellular Ca^2+^ signals. At this point a new, two-step “cross-talk” odorant transduction model was proposed (Duda and Sharma, 2009). In “step 1,” the odorant uroguanylin interacts with the receptor domain of ONE-GC, causing its minimal activation. The cyclic GMP generated in response to uroguanylin signal opens a few of the CNG3 channels leading to some increase in [Ca^2+^]**_i_ in the odorant receptor cell. In “step 2,” the membrane bound NCδ and GCAP1 sense the increase in [Ca^2+^]**_i_ and in their Ca^2+^-bound states fully activate ONE-GC triggering maximal influx of Ca^2+^and depolarization of the olfactory receptor cell membrane.

The odorant receptor ONE-GC linkage with the intracellular free Ca^2+^ signals brought forth a question whether this cyclase is a unique case of dually regulated membrane guanylate cyclase. Latest studies from our laboratory demonstrate that it is not. The nascent member of the hormone receptor subfamily, the ANF-RGC, is also responsive to Ca^2+^ (Duda et al., 2012a,b) and NCδ is the sensor protein which communicates the Ca^2+^ signal to ANF-RGC and in the Ca^2+^-bound state, activates ANF-RGC activity. The two modes of ANF-RGC activation, hormonal and Ca^2+^-dependent, engage their specific and independent pathways of transmitting the stimulatory signals to the catalytic domain. The end-product, however, is common, the second messenger cyclic GMP. Because of the individual signaling mechanisms involved in Ca^2+^ and hormonal signaling their net effects are multiplicative. Hence, in terms of the original sub-classification, the present day knowledge is that at least two cyclases, ANF-RGC and ONE-GC, are hybrids sensing both hormonal and Ca^2+^ signals.

The last cloned membrane guanylate cyclase was GC-G. Until today, the information of its function is very scarce. It was suggested that the mouse form of GC-G is selectively expressed in the sperm and may be involved in the process of capacitation (Kuhn et al., 2004). Other reports suggest that the cyclase is expressed in Grueneberg ganglion olfactory subsystem where it is responding to CO_2_ (Chao et al., 2010) or to cool ambient temperature indicating its role in thermo-sensation (Mamasuew et al., 2008).

The following discussion is exclusively devoted to the ANF-RGC. It was the first template model, which established that cyclic GMP is a *bona fide* hormonal second messenger. The ongoing studies define the second ANF-RGC transduction model, Ca^2+^-modulated signaling. It makes ANF-RGC a bimodal switch, hormonal and Ca^2+^. An additional significant part of the review is coverage of the manner in which these two models have now begun to explain the biochemical principles of cardiovascular, renal and endocrine homeostasis with special emphasis on blood pressure regulation.

## HORMONAL SIGNALING OF ANF-RGC ACTIVATION

### BINDING OF LIGAND-HORMONE ANF

Although intuitively expected, the first experimental evidence that the ANF binding site in ANF-RGC is located within the extracellular domain came first from the site-directed and deletion mutagenesis studies. A mutant ANF-RGC was cloned from rat adrenal cDNA library (Duda et al., 1991) and termed GCα. Its sequence differed from ANF-RGC by two amino acid substitutions Q^338^ → H and L^364^ → P which resulted from single nucleotide mutations, CAG → CAC and CTG → CCG, respectively. Expressed in heterologous system of COS cells, GCα was properly located in the plasma membrane and exhibited basal guanylate cyclase activity; it however, neither bound nor responded to ANF or other natriuretic peptides (Duda et al., 1991). These results demonstrated that the two amino acid substitutions exclusively abolished binding of ANF and therefore ANF signaling of the cyclase activity. Restoration by site-directed mutagenesis, glutamine at position 338 and leucine at position 364, reinstated ANF binding and ANF-dependent stimulation of the cyclase.

The site of ANF binding was further systematically analyzed (McNicoll et al., 1992, 1996; He et al., 1995; Misono, 2002). By cross-linking and proteinase digestion it was determined that the amino terminus of ANF is in contact with the region M^173^-F^188^ of ANF-RGC, and the C-terminus, with D^191^-R^198^ region. The fact that the identified contact sites were very close to each other was interpreted that the N- and C- termini of ANF interface distinct subunits of ANF-RGC homodimer. Importantly, it also implied that one ANF-RGC dimer binds one molecule of ANF.

Details of ANF binding to the ANF-RGC extracellular domain were unraveled by analyses of the extracellular domain of ANF-RGC co-crystallized with ANF (Ogawa et al., 2003, 2004). These analyses revealed that (1) the extracellular domain of ANF-RGC exists as a dimer in the head-to-head configuration; (2) one ANF-RGC dimer binds one molecule of ANF (2:1 complex); (3) the binding site in one monomer differs from that in the other - one monomer binds the N-terminal part of ANF and the other binds C-terminal part; (4) there is no preference in ANF binding to a specific monomer of the extracellular domain; bound ANF occurs in two alternate orientations of equal occurrence; (5) the membrane-distal portion of the extracellular domain contains chloride ion necessary for ANF binding.

### TRANSMEMBRANE MIGRATION OF THE ANF BINDING SIGNAL

With the exception of enterotoxin RGC, all membrane guanylate cyclases contain a hinge region juxtaposed to the N-terminal side of the transmembrane domain. This region contains two conserved cysteine residues separated from each other by 6–8 residues. In ANF-RGC these residues are Cys^423^ and Cys^432^ and were indicated as a critical structural motif in ANF signaling (Huo et al., 1999; Labrecque et al., 1999; Miyagi and Misono, 2000). There was however no consensus on the mechanism of its operation. Based on results with intact cells transfected with ANF-RGC cDNA it was proposed that the cysteines are involved in dimerization through the formation of an inter-chain S-S bridge (Huo et al., 1999; Labrecque et al., 2001) or, that they form an intra-chain disulfide bridge (Huo et al., 1999; Miyagi and Misono, 2000). Analyses of guanylate cyclase activity in isolated membranes of COS cell expressing C^423^S, C^432^S, and C^423^S, C^432^S mutants (Duda and Sharma, 2005) allowed to conclude that both C^423^ and C^432^ residues independently and equally control the catalytic activity of ANF-RGC and that activation of ANF-RGC does not involve interchain-disulfide bond formation. In the basal state, the disulfide motif keeps the ANF-RGC in its repressed catalytic state and ANF/ATP signaling brings it to the fully active state (Duda and Sharma, 2005).

A model of hormone-induced rotation has been proposed to explain how the extracellular signal is transmitted through the hinge region to the intracellular domain of ANF-RGC (Ogawa et al., 2004; Misono et al., 2005). Binding of ANF causes a twist motion of both ANF-RGC monomers centered on a support point close to the bound ANF. This twist motion translocates the juxtamembrane domains of both monomers with only minimal change in the distance between them. This movement constitutes a signal, which is transmitted through the transmembrane domain. Rotation of the transmembrane domain by 40 degrees occurs in response to ANF binding (Parat et al., 2010).

### PASSAGE OF THE ANF SIGNAL THROUGH THE INTRACELLULAR DOMAIN

#### ANF signaling requires ATP

Early studies, before the biochemical characterization of ANF-RGC, demonstrated that activation by ANF is significantly amplified by ATP in the guanylate cyclase activation (Kurose et al., 1987; Cole et al., 1989; Chang et al., 1990; Larose et al., 1991; reviewed in Sharma, 2002; Duda et al., 2005). Subsequently, it was demonstrated that ATP is obligatory for ANF-dependent activation of ANF-RGC (Chinkers et al., 1991; Marala et al., 1991; Goraczniak et al., 1992; Wong et al., 1995; reviewed in Duda et al., 2005). Because the ATP effect was mimicked by its non-hydrolyzable analogs, ATPγS and AMP-PNP, it was suggested that ATP acts as an allosteric regulator (Chinkers et al., 1991; Marala et al., 1991; Goraczniak et al., 1992; Wong et al., 1995; reviewed in Duda et al., 2005). Shortly thereafter it was shown that ATP is also obligatory for the CNP-dependent stimulation of CNP-RGC (Duda et al., 1993a,b). Thus, it appears that the requirement of ATP is a common attribute of natriuretic peptide RGC signaling.

Studies with ANF-RGC deletion mutants revealed that the ATP regulated region resides in the intracellular portion of ANF-RGC between the transmembrane and the C-terminal catalytic domain (Chinkers and Garbers, 1989; Goraczniak et al., 1992). This region was named the kinase homology (or kinase-like domain; KHD or K-LD) because of its sequence similarity with the corresponding domains of tyrosine kinases (Chinkers et al., 1989; Chinkers and Garbers, 1989; Duda et al., 1991). A model for the ATP function was proposed that “binding of ANP to the extracellular domain of its receptor initiates a conformational change in the protein K-LD, resulting in de-repression of guanylate cyclase activity” (Chinkers and Garbers, 1989). The central idea behind the model was that KHD in native ANF-RGC suppresses the catalytic module activity; ANF functions by relieving this suppression. Study from our group using two KHD deletion mutants, Δ506–677 and Δ555–762, negated this model and proposed an alternative one where ATP via ATP regulated module (ARM) potentiates the hormonal signal “(1) the signal is caused by the binding of the hormone to the receptor site; (2) there is a transmembrane migration of the signal; (3) the signal is potentiated by ATP at ARM; and (4) the amplified signal is finally transduced at the catalytic site” (Goraczniak et al., 1992).

#### ATP allosteric effect and the ARM domain

Within the KHD ANF-RGC contains a sequence, G^503^-X-G^505^-X-X-X-G^509^ (Goraczniak et al., 1992) which is a modified form of the protein kinases’ nucleotide-binding consensus sequence G-X-G-X-X-G necessary for kinase activity (Wierenga and Hol, 1983; Hanks et al., 1988; Bratová et al., 2005). This motif was probed for its significance in ATP function in ANF signaling. Through analyses of a series of deletion and point mutants it was determined that the G^503^-X-G^505^-X-X-X-G^509^ motif is critical for the ATP function (Goraczniak et al., 1992; Duda et al., 1993a; Duda and Sharma, 1995; reviewed in Sharma, 2002; Duda et al., 2005). Although it is not involved in ATP binding it maintains the steric arrangements to fit the ATP molecule. For this reason, the motif has been named ARM (Goraczniak et al., 1992) and the KHD, where the ARM resides, was termed the ARM domain (Duda et al., 2001b; Sharma et al., 2001). To get an insight into the mechanism of ATP function, the structure of the ARM domain was simulated through computer-assisted homology based modeling (Duda et al., 2001b; Sharma et al., 2001; PDB ID 1T53). The basic structural features of the model have been experimentally validated through point mutation/expression studies (Duda et al., 2001b; Sharma et al., 2001; reviewed in Sharma, 2002; Duda et al., 2005).

***Spatial determinants of the ARM domain***. The domain comprises amino acid residues 496–771, which are arranged into two lobes: the smaller, N-terminal (91 aa residues: 496–586), and the larger, C-terminal (185 aa residues: 587–771; Duda et al., 2001b). Four antiparallel β strands and one α helix form the smaller lobe; the larger lobe is predominantly helical, composed of eight α-helices and two β strands (Duda et al., 2001b; Sharma et al., 2001). The ARM motif, G^503^-X-G^505^-X-X-X-G^509^, is located within the smaller lobe.

***The ATP-binding pocket***. In order to identify the ARM domain residues potentially involved in ATP binding the model of this domain in its ATP-bound form was analyzed (Duda et al., 2005). A radius of 4 Å from the ATP molecule was chosen as the limiting distance for the electrostatic, hydrogen or van der Waals’ interactions. Two sets of residues were identified (Figure 4 in reference Duda et al., 2005): (1) those forming the floor of the ATP binding pocket; (2) those surrounding ATP molecule. The residues in the first set are: G^503^, R^504^, G^505^, S^506^, N^507^, Y^508^, and G^509^. These residues have no direct chemical interaction with ATP; they, however, provide necessary space to accommodate the ATP molecule. The residues in the second set surround the individual components of ATP, the adenine ring, the ribose ring and the triphosphate moiety. The adenine ring is surrounded by L^511^, T^513^, T^514^, E^515^, Q^517^, A^533^, T^580^, E^581^, C^583^, V^635^, and T^645^ and L^511^, T^513^, and C^583^ are within a distance shorter than 3 Å.

G^503^, L^511^, T^513^, T^514^, G^580^, S^587^, D^590^surround the ribose ring with L^511^ and T^514^ located within 2.5 Å radius from the ribose. The phosphate groups are surrounded by R^504^, G^505^, L^511^, K^535^, N^633^, D^646^, and K^535^ is the nearest residue (2.6 Å) forming a hydrogen bond with the phosphate and has been shown to be critical for ATP regulation of ANF-dependent ANF-RGC activity; D^646^ interacts with the triphosphate group of ATP through the formation of a coordinate bond with the metal ion Mg^2+^.

***Interaction of ATP with the ARM domain***. Kinetics of ATP binding to the ARM domain was determined through SPR spectroscopy (Burczynska et al., 2007). AMP-PNP, the non-hydrolyzable analog of ATP was used for the binding studies. Half-maximal binding (EC_50_) occurred when the concentration of AMP-PNP was ~0.2 mM and the calculated *K*_D_ value was 0.21 mM. Similar results (EC_50_ value of 0.15 mM and *K*_D_ of 0.13 mM) were obtained when 8-azido-ATP was used.

The model-predicted ATP binding pocket (*vide supra*) was authenticated experimentally by cross-linking of the purified isolated ARM domain protein with 8-azido-ATP, trypsin digestion of the cross-linked product and sequencing of the resulting peptides (Burczynska et al., 2007). Three peptides were found to be photoaffinity modified. The longest modified peptide was identified as G^614^MLFLHNG-AICSHGNLKSSNCVVDGR^639^and the shortest as S^631^SNC^634^V^635^VDGR. In all three peptides Cys^634^was modified indicating that this residue was the closest to the azido group. The G^614^–R^639^ fragment contains six ATP-binding pocket-predicted residues (Duda et al., 2005): S^625^, K^630^, S^631^, S^632^, N^633^, and V^635^; and the shortest S^631^–R^639^ fragment contains three residues: S^631^, N^633^, and V^635^(Duda et al., 2005). The refined ARM model (Duda et al., 2005) predicts that V^635^ is within a 4 Å radius from the adenine ring; cross-linking of the neighboring residue to C^634^ validates that these residues are indeed the closest to the C-8 of the adenine ring.

***ATP binding dependent changes in the ARM domain.*** If ATP binding to the ARM domain were to serve signaling purposes, it should induce structural changes that ultimately would signal activation of the catalytic domain. Comparative analyses of the ARM domain models in the apo and ATP-bound states identified such changes. They involve rotations of the β strands within the smaller lobe as well as movements of the β strands and α helices in the larger lobe (Duda et al., 2001b; Sharma et al., 2001; reviewed in Duda et al., 2005). Consequences of two such changes appear to be of particular importance: first, of the β1, β2 strands in the smaller lobe, and the second, of the EF and F helices in the larger lobe.

The β1 and β2 strands and the loop connecting them encompasses the G^503^-X-G^505^-X-X-X-G^509^ motif (Duda et al., 2001b; Sharma et al., 2001; reviewed in Duda et al., 2005), which is meshed in and flanked by six phosphorylation sites, S^497^, T^500^, S^502^, S^506^, S^510^, T^513^ (Potter and Hunter, 1998a, 1999a). The present consensus is that “phosphorylation of KHD is absolutely required for hormone-dependent activation of NPR-A” (Potter and Hunter, 1998a,b, 1999b). In this concept, hypothetical protein kinase and protein phosphatase co-exist with ANF-RGC (Foster and Garbers, 1998). Until now, however, neither the kinase nor the phosphatase has been identified.

**FIGURE 1:**
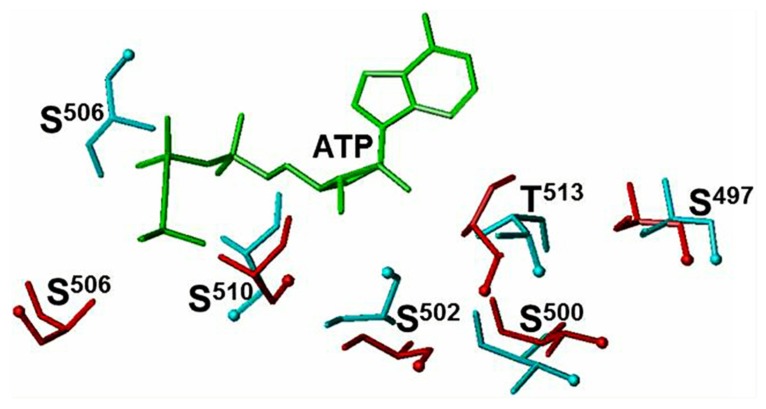
**ATP binding to the ARM domain affects the conformation of the six phosphorylable residues.** The conformation of the six phosphorylated residues is shown before (cyan) and after (red) ATP binding. The ATP molecule is shown in green. The positions of the OH groups are indicated by cyan and red balls (reproduced with permission from ref. Duda et al., 2011).

Comparison of the ATP-free and ATP-bound ARM domain models allows explaining how ATP binding makes it possible for the serine and threonine residues to undergo phosphorylation. After ATP binds to the ARM domain, the β1, β2 strands, and the loop between them shift by ~3–4 Å and rotate by ~15° (Duda et al., 2001b; Sharma et al., 2001; reviewed in Duda et al., 2005). This movement triggers reorientation of the serine and threonine residues (**Figure 1**, red colored residues) causing the side chains and the OH groups of T^500^, S^502^, S^506^, and T^513^ becoming directed toward the protein surface (**Figure 1**, compare the positions of the cyan- and red-colored OH groups). The change in the positions of the side chains is most drastic for the S^502^ and S^506^ residues. Although upon ATP binding there is no toward the surface reorientation of the S^497^ and S^510^ OH groups, the entire residues are shifted toward the surface (**Figure 1**). This structural rearrangement permits the hypothetical protein kinase to access the side chains of the residues and transfer the phosphate group (Duda et al., 2011).

**FIGURE 2:**
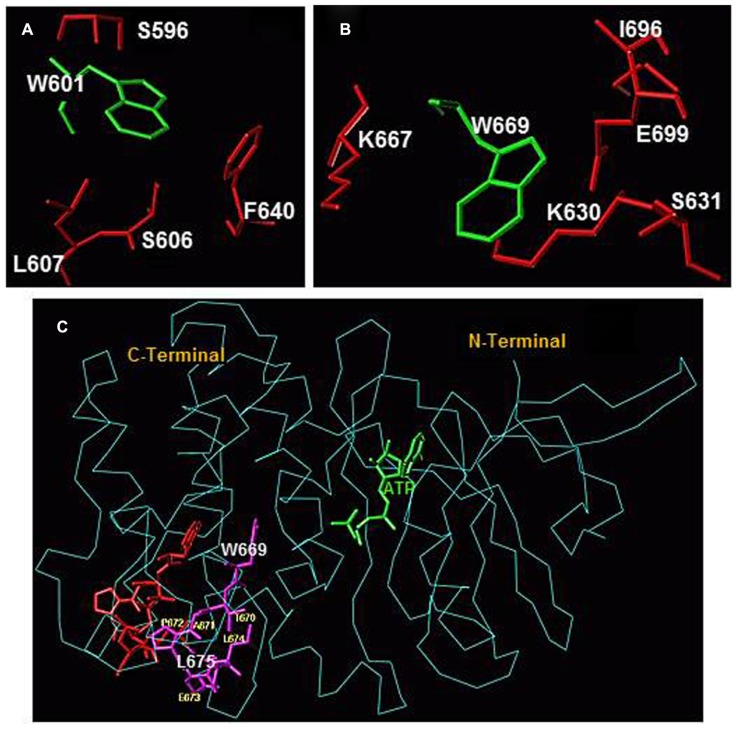
**(A)** Amino acid residues surrounding W^601^ and **(B)** amino acid residues surrounding W^669^ within the ARM domain. Amino acid residues depicted in red are located within a 4 Å sphere from the respective tryptophan residue (green). **(C) **Conformational changes within the ^669^WTAPELL^675^ motif induced by ATP binding to the ARM domain. The backbone structure of the ATP-bound ARM domain is shown in cyan and the ATP molecule is in green. The ^669^WTAPELL^675^ motif is shown in magenta color. Apo structure of the ARM domain was superimposed on the ATP-bound form to assess the relative, ATP binding induced, conformational changes. For clarity, only the ^669^WTAPELL^675^ motif (shown in red) of the apo-enzyme is visible. ATP binding results in a more compact structure of the ARM domain: the W^669^ side chain moves toward the ATP binding pocket while the side chains of T^670^, E^673^, L^674^, and L^675^ move toward the protein surface [compare the orientation of side chain of these amino acid residues before (in red) and after (in magenta) ATP binding; fonts for W^669^ and L^675^ residues are increased for better visibility]. This movement changes the surface properties of the ARM domain. The movement toward the surface of the protein is poised to facilitate interaction of this amino acid stretch with subsequent transduction motif, possibly within the catalytic domain, propagation of the ANF/ATP binding signal and activation of the catalytic domain (reproduced with permission from ref. Duda et al., 2009).

Another important result of ATP binding on the dynamics within the ARM domain is the translocation within the larger lobe of two helices, EF and F. Their movement was characterized by analyzing the ATP-dependent changes in the fluorescence intensity and wavelength of two tryptophan residues W^601^ and W^669^ (Duda et al., 2009). Both residues reside in the larger lobe of the ARM domain, outside the ATP-binding pocket. The W^601^ residue is a part of the helix E structure (Duda et al., 2001b, 2005), its side-chain is oriented toward the protein surface, and it is flanked by several hydrophobic residues, F^640^, L^607^, S^606^, and S^596^ (**Figure 2A**). These structural features define the fluorescence λ_max_ of W^601^ at 332 nm. W^669^ residue is located at the end of a loop connecting β8 strand and EF helix (Duda et al., 2009); it is a part of a conserved hydrophobic motif, ^669^WTAPELL^675^. The side chain of W^669^ is flanked by the polar residues: E^699^, S^631^, K^667^, K^630^, and L^696^ (**Figure 2B**). This environment causes red shifts in the fluorescence λ_max_ of W^669^ to 345 nm.

Superimposition of the ATP-free and the ATP-bound forms indicates that ATP binding induces contraction of the entire ARM domain and affects the orientation and environment of W^669^. The contraction results in shortening of the distance between W^669 ^and the ATP binding pocket (**Figure 2C**). ATP binding also causes reorientation of the W^669^ side chain. It turns and becomes more shielded by the surrounding amino acids. Turning of the W^669^ side chain pushes the remainder of the motif, ^670^TAPELL^675^, to the surface resulting in its exposure. In general, the movement of a hydrophobic motif toward the surface of the protein indicates its readiness for interaction. For the ^669^WTAPELL^675^ motif it was proposed that it interacts with subsequent transduction motif, possibly within the catalytic domain, propagates the ANF/ATP binding signal and activates the catalytic domain (Duda et al., 2009).

Based on the studies narrated above a model for ANF/ATP signaling of ANF-RGC activity was proposed: The ANF signal originates by the binding of one molecule of ANF to the extracellular dimer domain of ANF-RGC (Ogawa et al., 2004, 2009). The binding modifies the juxtamembrane region where the disulfide ^423^Cys-Cys^432^ structural motif is a key element in this modification (Ogawa et al., 2004, 2009; Duda and Sharma, 2005). The signal twists the transmembrane domain (Parat et al., 2010), induces a structural change in the ARM domain, and prepares it for the ATP activation. ARM domain binds ATP to its pocket what leads to a cascade of temporal and spatial changes (Duda et al., 2001b; Sharma et al., 2001; reviewed in Duda et al., 2005). They result in (1) exposure of the hydrophobic ^669^WTAPELL^675^ motif which directly (or indirectly) interacts with the catalytic domain causing its partial activation; and (2) exposure and phosphorylation of six serine, threonine residues and full activation of ANF-RGC. Concomitantly, phosphorylation converts ATP binding site from the high to low affinity, ATP dissociates and ANF-RGC returns to its ground state (Duda et al., 2011).

### CATALYTIC DOMAIN

When more then 20 years ago an ANF RGC was purified to homogeneity and shown to contain the ANF binding and cyclic GMP forming activities on the same protein chain, the authors proposed a topological model for the transmembrane receptor enzyme in which the receptor part was extracellular and the cyclase catalytic domain was intracellular (Sharma et al., 1988a,b). The cloning studies confirmed this prediction. Alignment of the deduced amino acid sequences of the cloned guanylate cyclases indicated that the catalytic domain is located at the C-terminus of ANF-RGC and comprises 239 amino acid residues (Chinkers et al., 1989; Duda et al., 1991). This prediction was tested experimentally (Thorpe and Morkin, 1990; Thorpe et al., 1996). The carboxy terminal 293 amino acids fragment of ANF-RGC was expressed as a soluble protein and shown to exhibit guanylate cyclase activity. These results were in agreement with studies that determined through radiation inactivation experiments that the ANF-RGC fragment containing cyclase activity has a molecular weight of 32 ± 8 kDa (Tremblay et al., 1991). Further studies identified several residues within this region that appeared to be critical for the guanylate cyclase activity: L^817^ (Miao et al., 1995), D^877^, K^887^, D^893^, G^900^, H^909^, R^940^, and H^944^ (Thompson and Garbers, 1995). Their individual mutations to Ala resulted in ANF-RGC mutants without detectable guanylate cyclase activity. In contrast to these residues, mutation of E^974^ to Ala resulted in a hyperactive ANF-RGC mutant (Wedel et al., 1997). Based on these results it was concluded that the indicated residues are located within or close to the catalytic center or are critical for the proper folding of the catalytic center.

In the absence of a crystal structure of any membrane guanylate cyclase catalytic domain a model of the catalytic center of retGC-1, a mammalian membrane guanylate cyclase expressed in the retina, also known as ROS-GC1 was proposed (Liu et al., 1997). The model was built based on homology with the catalytic center of the adenylate cyclase. The modeling studies allowed identification of the critical residues constituting the catalytic core. Because the catalytic domains of all membrane guanylate cyclases are highly conserved (>95% of sequence identity), by homology substitution, the critical residues of the ANF-RGC catalytic center have been identified and they are described below.

The catalytic center is homodimeric. There are two GTP binding sites. Each GTP molecule interacts with both monomers. In the following description of the ANF-RGC catalytic center the monomers are labeled “A” and “B” and the location of each residue within a monomer A or B is indicated. Numbering of amino acids is according to the mature protein (Duda et al., 1991). R^959^(B) and C^961^(B) interact with guanine’s O6; second NH_2_ group of R^959^(B) interacts with guanine’s N1; the carboxy group of E^889^(B) interacts with R^959^(B) and through it with N1; G^964^(B) interacts with N7; T^854^(A), D^893^(A) and N^968^(B) form hydrogen bonds with ribose’s OH (3′) group; R^972^(B binds α phosphate group; R^940^(A) binds β phosphate group; E^974^(A) through Mg^2+^ interacts with β phosphate group; and S^971^(A) binds γ phosphate group. Specificity toward GTP is determined by E^889^ and C^961^ of ANF-RGC. This conclusion is drawn based on studies showing that in ROS-GC1 mutation of the corresponding E to K and C to D converts guanylate cyclase activity into adenylate cyclase activity (Tucker et al., 1998).

#### Mechanism of ANF-RGC activation – the role of the ^669^WTAPELL^675^ motif

An important feature of the model of ANF/ATP-dependent activation of ANF-RGC is the relay function of the ^669^WTAPELL^675^ motif. It switches between the ATP-bound ARM domain and activates the catalytic domain. It was proposed that this motif interacts with and signals the antiparallel homo-dimer of the catalytic domain to undergo conformational changes and form a functional catalytic center (Duda et al., 2009). The exact site within the catalytic domain which is targeted by the ^669^WTAPELL^675^ motif remains to be determined. Preliminary scanning experiments, however, suggest that the site may be located close to the E^889^, a residue critical for cyclase specificity toward GTP (Tucker et al., 1998).

If the elements of the model are correct and the ^669^WTAPELL^675^ motif is a switch, its deletion from the ANF-RGC should lead to a protein which binds ANF and ATP but is not able to respond to them with increased synthesis of cyclic GMP. This assumption was tested experimentally. An ANF-RGC mutant was created in which the ^669^WTAPELL^675^ motif was deleted by mutagenesis. Wild type ANF-RGC and the mutant were expressed individually in COS cells and their membranes were analyzed (1) by Western blot to determine the proteins’ levels of expression; (2) for basal guanylate cyclase activity; (3) for ANF binding; and (4) for K_M_ for the substrate GTP. The results demonstrated that (1) the wild type ANF-RGC and the ^669^WTAPELL^675^ deletion mutant were expressed to the same level as determined by Western blot; (2) the basal cyclase activity of the mutant, 20 pmol cGMP min^-1^ (mg protein)^-1^, was virtually identical to that of the wild type ANF-RGC, 21 pmol cGMP min^-1^ (mg protein) ^-1^; (3) their receptor activities were equal with the respective specific binding values of 9.7 ± 0.6 and 10.1 ± 0.9 pmol [^125^I]ANF/mg protein and (4) the K_M_ values for the substrate GTP, 614 μM for the wild type and 608 μM for the mutant protein were identical. Thus, the basal integrity of the protein remained intact despite the deletion (Duda et al., 2009).

When the mutant was exposed to increasing concentrations of ANF and ATP its cyclase activity remained practically unchanged whereas under identical conditions wild type ANF-RGC was stimulated over 6-fold (Duda et al., 2009, 2013) demonstrating that in line with the original expectations the catalytic domain does not increase cyclic GMP synthesis in the presence of ANF/ATP. This type of outcome could only be observed if the mutant protein did not bind ANF and/or ATP or the catalytic domain lost its cyclase ability. Both of these possibilities were put to rest as the mutant and the wild type cyclase did not differ in ANF and ATP binding nor in enzymatic activity (*vide supra* and ref. Duda et al., 2009). Therefore the only explanation for the unchanged level of cyclic GMP synthesis is that the ^669^WTAPELL^675^ deletion mutant is not able to respond to ANF and ATP. This leads to just one logical conclusion that in the absence of the ^669^WTAPELL^675^ motif the ANF/ATP binding information is not transmitted to the catalytic domain. Thus, the ^669^WTAPELL^675^ motif is the critical transmitter of the ATP-potentiated ANF signal to the catalytic domain where it is translated into generation of cyclic GMP.

## Ca^2+^ SIGNALING OF ANF-RGC ACTIVITY

### NEURONAL CALCIUM SENSOR NEUROCALCIN δ

Neurocalcin δ belongs to a distinct subfamily of neuronal calcium sensor proteins (NCS) together with visinin-like proteins (VILIPs) and hippocalcin. They all are acylated at the N-terminus by myristic acid and undergo a classical calcium-myristoyl switch (Ladant, 1995), e.g., they bury the myristoyl group in a hydrophobic pocket in Ca^2+-^free form and expose it in Ca^2+^-bound form, as first observed and described for recoverin (Zozulya and Stryer, 1992). Exposure of myristoyl group enables the protein association with the cell membrane. However, once it binds, in a Ca^2+^-dependent fashion, to the membrane phospholipids, even after removing Ca^2+^ by the addition of EGTA part of it remains membrane bound (Krishnan et al., 2004). Although NCδ is primarily expressed in neuronal tissues, its expression in the periphery is also observed.

Functionally, NCδ has been linked to receptor endocytosis through interaction with α- and β-clathrin and β-adaptin (Ivings et al., 2002), trafficking and membrane delivery of glutamate receptors of the kainate type (Coussen and Mulle, 2006), and due to its Ca^2+^-dependent affinity for S100B protein and tubulin β-chain (Okazaki et al., 1995), with microtubule assembly (Iino et al., 1995). In the sensory and sensory-linked neurons, the presence of NCδ has been found in the inner plexiform layer of the retina, e.g., in the amacrine and ganglion cells (Krishnan et al., 2004), olfactory sensory neurons (Duda et al., 2001a, 2004) and recently, it has been identified in type II cells of mouse circumvallate taste papillae, indicating its possible role in gustatory transduction (Rebello et al., 2011).

A relatively newly identified function of NCδ is its Ca^2+^-dependent modulation of the activities of membrane guanylate cyclases ROS-GC1 in the retina and ONE-GC, in the olfactory neuroepithelium (Duda et al., 2001b, 2004; Krishnan et al., 2004). In these tissues NCδ co-localizes with its respective target cyclases. The exact physiological significance of the ROS-GC1- NCδ signaling system in the retinal neurons is not known yet. It can be, however, safely stated that the pathway is not present in the rod and cone outer segments, thus is not linked with the phototransduction machinery. The system has been localized to the lower strata of the inner plexiform layer and to a subpopulation of ganglion cells (Krishnan et al., 2004).

In the olfactory neuroepithelium NCδ serves as a Ca^2+^ sensor component of the two-step odorant uroguanylin signaling machinery. This signaling mechanism was proposed to be initiated by uroguanylin interaction with the extracellular receptor domain of ONE-GC (Leinders-Zufall et al., 2007; Duda and Sharma, 2008; reviewed in Zufall and Munger, 2010; Sharma and Duda, 2010). This interaction leads to partial activation of ONE-GC, generation of small amount of cyclic GMP, partial opening of cyclic GMP-gated channel and influx of Ca^2+^ into the olfactory receptor neuron. In the next step, Ca^2+^ binds to NCδ which, then fully activates ONE-GC (Duda and Sharma, 2009).

### FREE CA^2+^ SIGNALS ANF-RGC ACTIVATION

In the course of mapping the NCδ targeted site on ROS-GC1 to which it binds and transmits the Ca^2+^ signal to the catalytic domain for signal translation into the generation of cyclic GMP, our group made a remarkable observation that NCδ binds directly to the catalytic domain and, thereby, activates ROS-GC1 (Venkataraman et al., 2008). Protein database comparison shows sequence conservation of the catalytic domain in the membrane guanylate cyclase family hinting at a possibility that other membrane guanylate cyclases might be activated by Ca^2+^ via NCδ as well. To test this possibility, ANF-RGC membrane guanylate cyclase, for which the only established signal transduction mechanism was through ANF/ATP (*vide supra*), was chosen. And indeed, in a recombinant system, myristoylated NCδ stimulated ANF-RGC activity in a dose- and Ca^2+^-dependent manner; 0.5 μM Ca^2+^ and 0.5 μM NCδ triggered half-maximal activation of ANF-RGC (Duda et al., 2012a,b). These results for the first time demonstrated that ANF-RGC activity is dually regulated, by peptide hormones ANF and BNP, and by Ca^2+^, thus, at least *in vitro* the cyclase was deemed a bimodal signal transducer.

#### Myristoylated dimeric form of neurocalcin δ is the transmitter of the Ca^2+^ signal

Since NCδ belongs to the family of NCS and myristoylation at N-terminus is a characteristic feature of a majority, but not all, of these proteins important for their cellular function, it was necessary to check whether myristoylation was required for NCδ to transmit the Ca^2+^ signal for ANF-RGC activation. Reconstitution experiments of ANF-RGC with both myristoylated and non-myristoylated NCδ showed that only the myristoylated NCδ stimulated the cyclase activity whereas the non-myristoylated form was ineffectual (Duda et al., 2012b). Importantly, both forms exhibited comparable affinity for ANF-RGC. In addition to activating ANF-RGC myristoylated NCδ also lowered the cyclase’s K_M_ for substrate GTP and increased its catalytic efficiency, *k*_cat_, from 6.5 ± 0.3 to 41.4 ± 0.5 pmol cyclic GMP/s.

The biochemical and homology based modeling studies indicate that the secondary structure of the functional form of all membrane guanylate cyclases is homodimeric (Rondeau et al., 1995; Liu et al., 1997; Venkataraman et al., 2008). The contact points for their homo-dimeric formation reside in their extracellular domain (Misono et al., 2011) and in the intracellular domain within the highly conserved dimerization domain (Wilson and Chinkers, 1995) and core catalytic core domain (Venkataraman et al., 2008). The X-ray crystallographic studies have demonstrated that NCδ also exists as a dimer (Vijay-Kumar and Kumar, 1999). Thus, it was reasonable to expect that the Ca^2+^-modulated functional unit is NCδ dimer and ANF-RGC dimer.

This expectation was validated experimentally. When monomeric and dimeric forms of myristoylated NCδ were individually tested in reconstitution experiments for their abilities to stimulate ANF-RGC catalytic activity only the dimer was effective (Duda et al., 2012b). The stimulation by the monomeric form was only marginal, possibly resulting from spontaneous dimerization of the monomers when higher NCδ concentrations were used (Duda et al., 2012b). Thus indeed the functional Ca^2+^ signal transduction unit is composed of one NCδ dimer and one ANF-RGC dimer.

#### Neurocalcin δ targets directly the catalytic domain of ANF-RGC

The ANF-RGC fragment, aa 788–1029, encompassing the core catalytic domain, I^820^–G^1029^, was expressed as a soluble protein and tested for its basal and Ca^2+^-dependent modulated *via* NCδ activities. The protein exhibited intrinsic guanylate cyclase activity (18 ± 4 pmol cyclic GMP min^-1^ mg protein^-1^). This activity increased when Ca^2+^ and NCδ were added, and the increase was Ca^2+^ and NCδ-dose dependent (Duda et al., 2012b). Interestingly, the estimated EC_50_ for NCδ was comparable to that determined for full-length ANF-RGC strongly supporting the expectation that the NCδ signaling site resides within the catalytic domain.

The NCδ targeted site on ROS-GC1 was mapped to the aa segment V^837^–L^858^. The corresponding site on ANF-RGC, ^849^DIVGFTALSAESTPMQVVTLLMQ^871^, has 70% sequence conservation in comparison with ROS-GC1. When a synthetic peptide of this sequence was used in a functional interference experiment almost complete inhibition of the NCδ-stimulated ANF-RGC activity at 200 μM with an IC_50_ value of 80 μM was observed (Duda et al., 2012b). Because a scrambled peptide did not exhibit any inhibitory effect it was justified to conclude that the ANF-RGC region ^849^DIVGFTALSAESTPMQVVTLLMQ^871^ mediates NCδ-dependent Ca^2+^ stimulation of ANF-RGC activity. This region is a part of the core catalytic domain and common to the corresponding sites in other membrane guanylate cyclases^28^, it has a secondary structure of helix-loop-helix and is acidic in nature with a pI of 3.37 (Duda et al., 2012b).

#### The effects of ANF/ATP and Ca^2+^-neurocalcin δ signaling of ANF-RGC activity are multiplicative

To determine the liaison between ANF/ATP and Ca^2+^-NCδ signaling modes, ANF-RGC activity was analyzed first in the presence of 1 μM Ca^2+^ and 2 μM myristoylated NCδ and then with increasing concentrations, ranging from 10^-11^ M to 10^-6^ M, of ANF and constant 0.8 mM ATP. The presence of Ca^2+^ and NCδ resulted in stimulation of the cyclase activity approx. 3.5-fold above the basal activity. Addition of ANF and ATP resulted in additional 4.5-fold stimulation, demonstrating that the Ca^2+^-NCδ and ANF/ATP effects are multiplicative (Duda et al., 2012b). It is noteworthy that in the absence of Ca^2+^ in the reaction mixture only the ANF/ATP-dependent stimulation of ANF-RGC activity was observed.

The preceding narration on the mechanisms involved in the hormone- or Ca^2+^-NCδ-dependent stimulation of ANF-RGC activity explains the independence and multiplicativeness of the ANF and Ca^2+^-NCδ signals in the activation of ANF-RGC. It is based on our evolving conceptual scheme whose central idea is that the functional specificity of a guanylate cyclase is determined by the structure of its modular blocks. The structural motif, ^669^WTAPELL^675^ is involved in transmitting the ANF signal to the catalytic domain (Duda et al., 2009) but it is not involved in transmitting the Ca^2+^ signal to the catalytic domain; instead the ^849^DIVGFTALSAESTPMQVVTLLMQ^871^ motif is involved (Duda et al., 2012b).

## PHYSIOLOGICAL VALIDATION OF THE TWO SIGNALING MECHANISMS OF THE ANF-RGC ACTIVATION

**FIGURE 3:**
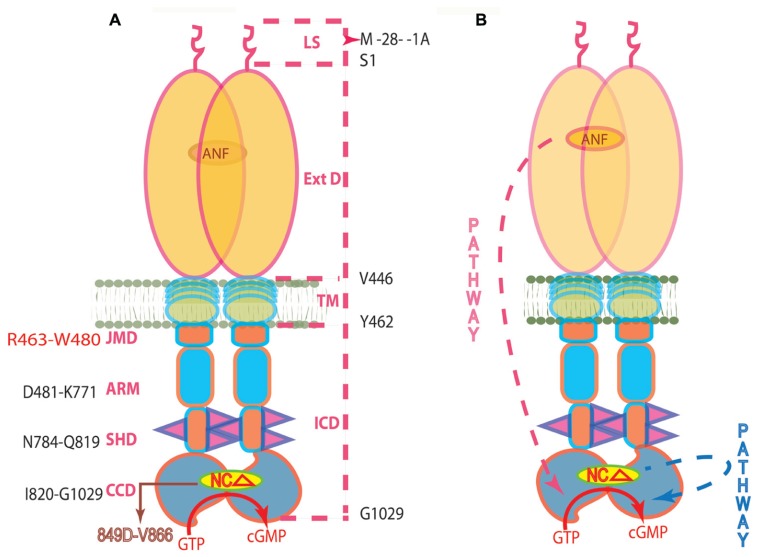
**(A)** Topography of ANF-RGC. The dashed lines on the right show the boundaries of: LS, leader sequence; ExtD, extracellular domain; TM, transmembrane domain; ICD, intracellular domain. The functional domains in ICD, their names and the aa constituting their boundaries are indicated at the left: JMD, juxtamembrane domain; ARM, the ATP regulated module; SHD-signaling helix domain; CCD-core catalytic domain. The site targeted by NCδ (encircled) is within CCD. **(B) **The signaling pathways of ANF and of NCδ are independent. The trajectory of the ANF pathway is in red dashed arrow. From the ExtD, it passes through the TM, ARM and SHD in its course to CCD. The trajectory of the NCδ pathway (in blue dashed arrow) is within the CCD. The CCD exists as an antiparallel homodimer (reproduced with permission from ref. Duda et al., 2012b).

The two described above signaling mechanisms of ANF-RGC activation are depicted in **Figure 3**. It is obvious from their description that they were developed based solely on biochemical observations. And although biochemically well-proven, they would have to be considered only hypothetical until their validity was confirmed *in vivo*. And this validation is narrated below.

### SIGNALING THROUGH THE ^669^WTAPELL^675^ MOTIF

In an *in vitro* reconstituted system the ^669^WTAPELL^675^ motif is critical for the transmission of the ANF/ATP signal and activation of the ANF-RGC catalytic domain and its absence results in unresponsiveness of the mutated ANF-RGC to the signal so if the same happens *in vivo*, an animal model in which the sequence coding for the ^669^WTAPELL^675^ motif is deleted from the ANF-RGC gene should be unresponsive to ANF and therefore exhibit all physiological consequences of this unresponsiveness. Following this logic, the ^669^WTAPELL^675^ sequence was deleted in genetically altered mice (Duda et al., 2013). It is worth mentioning that up till now it is the only ANF-RGC domain-specific genetically modified animal model.

In agreement with the *in vitro* determinations, basal ANF-RGC activity in the membranes of the heart, adrenal gland and kidney, the primary tissues where ANF-RGC is expressed, was practically indistinguishable between the wild type control (ANF-RGC^669^WTAPELL^675+/+^), heterozygous (ANF-RGC^669^WTAPELL^675+/-^) and homozygous (ANF-RGC^669^WTAPELL^675-/-^) mice (Duda et al., 2013). However, the mutated cyclase expressed in these tissues did not respond to ANF/ATP stimulation. The cyclase activity in the membranes of the homozygous mice was the same when assayed in the presence of 10^-7^ M ANF and 0.8 mM ATP as when assayed in their absence. Interestingly, for the heterozygous mice, the cyclase remained responsive to ANF/ATP but the maximal achieved activity oscillated around 50% of the wild type values, clearly showing that in the heterozygous mice where the product of one ANF-RGC gene copy is of the wild type and the other, of the deletion-mutated cyclase, only the wild type ANF-RGC is responsive to ANF/ATP and the mutant is not (Duda et al., 2013). Thus, the *in vitro* activity characteristics were the mirror images of these *in vivo*.

Almost identical values of basal guanylate cyclase activity in various tissues of the ^669^WTAPELL^675^ targeted mice and their isogenic controls implied that the expression of the mutant-cyclase must not differ from the expression of the wild type cyclase. This implication was confirmed by the results of immunocytochemical analyses. Side-by-side immunostaining with immunopurified anti ANF-RGC antibody resulted in identical images of the kidneys and adrenal glands of the wild type, heterozygous and homozygous ^669^WTAPELL^675^ targeted mice. Also, visual examination of the stained sections as well as of the respective differential interference contrast images indicated that the targeting of the ^669^WTAPELL^675^ motif does not change the integrity of these tissues (Duda et al., 2013).

The main function of ANF/ANF-RGC signaling system is to regulate natriuresis, diuresis, vasodilation, and prevent cardiac and renal hypertrophy. It regulates blood volume homeostasis and blood pressure by off-setting the renin-angiotensin-aldosterone system (RAAS): inhibiting renal renin secretion and aldosterone synthesis in the adrenocortical zona glomerulosa (Burnett et al., 1984; Brenner et al., 1990; Maack, 1996; Olson et al., 1998; Aoki et al., 2000; Shi et al., 2001). Therefore, elimination of ANF-RGC signaling through knocking-out either ANF-RGC or ANF gene leads to increased blood pressure, cardiac, and renal hypertrophy, fibrosis, to name some of the related pathological conditions (Dubois et al., 2000; Kishimoto et al., 2001; Ellmers et al., 2007; Das et al., 2010; Ellis et al., 2011; Pandey, 2011; Wadei and Textor, 2012). Since the ^669^WTAPELL^675^ targeted mice have partially (heterozygous) and fully (homozygous) suppressed ANF-RGC response to ANF, but the basal cyclase activity is unaffected the critical question was: does this suppression lead to increased blood pressure and other physiological alterations in the genetically modified mice?

The answer obtained was clearly “yes.” In comparison with the wild type the blood pressure of the heterozygous mice increased by ~37% and of the homozygous mice by ~56%. The increase was statistically highly significant with *P* value < 0.005 (Duda et al., 2013). It rose from 102 ± 9 mm Hg for the wild type mice to 134 ± 17 mm Hg for the heterozygous and 159 ± 11 mm Hg for the homozygous mice. These values showed that the progressive elevation of blood pressure directly correlates with the number of mutated ANF-RGC gene copies.

Because volume homeostasis and blood pressure are influenced by the mineralocorticoid hormone aldosterone secreted by the cells of the adrenocortical zona glomerulosa and the function of the ANF/ANF-RGC system is to inhibit this synthesis the obvious next question was whether the increased blood pressure in the ^669^WTAPELL^675^ targeted mice is a consequence of the inability of the mutated ANF-RGC to transduce the ANF signal and to synthesize cyclic GMP sufficient quantities to inhibit aldosterone synthesis? A hint was provided by studies on ANF-RGC knockout mice showing that these mice had increased aldosterone level (Zhao et al., 2007).

The plasma aldosterone concentrations were measured in both types of genetically modified mice (hetero- and homozygous) and in their isogenic controls (wild type). The results demonstrated that, in comparison with the wild type mice, the plasma aldosterone concentration increased by approximately 40% (from 147 ± 12 to 204 ± 18 pg/ml) in the plasma of the heterozygous mice (*P* < 0.005) and by approximately 75% (up to 256 ± 22 pg/ml) in the plasma of the homozygous mice (*P* < 0.005) (Duda et al., 2013).

Chronic pressure overload is almost always accompanied by cardiac hypertrophy (reviewed in Katholi and Couri, 2011). The same is observed in the ^669^WTAPELL^675^ targeted mice. The ratio of the heart weight (in mg) to whole body weight (in g) measured in 12 weeks old mice was 5.6 ± 0.3 and 6.1 ± 0.5 for the ^669^WTAPELL^675(+/-)^ and ^669^WTAPELL^675(-/-)^ mice, respectively, whereas it was 5 ± 0.3 for the wild type mice (Duda et al., 2013). However, hypertension is not the only cause of myocardial hypertrophy. Using animal models it has been shown that antihypertensive drugs do not always ameliorate cardiac hypertrophy (Knowles et al., 2001) and that even without systemic hypertension cardiac hypertrophy may occur (Oliver et al., 1997; Holtwick et al., 2003). Because deletion of the ^669^WTAPELL^675^ motif from ANF-RGC results in decreased synthesis of cyclic GMP it was hypothesized that cardiomyocytes of the ^669^WTAPELL^675^ targeted mice succumb to the insufficient quantities of cyclic GMP and normal inhibition of myocardial proliferative responses does not occur. Our ongoing studies indicate that indeed it is the case. The mice as young as 3 weeks of age (at weaning) exhibit significant cardiac hypertrophy. Which cyclic GMP-dependent signaling pathway involved in inhibition of myocardial proliferative responses is affected in these mice remains to be determined.

Taken together the biochemical and physiological evidence prove that the ^669^WTAPELL^675^ motif is critical for the ANF-RGC function as the transducer of the ANF/ATP signal.

### NEUROCALCIN δ Ca^2+^-DEPENDENT SIGNALING OF ANF-RGC

To demonstrate that the ANF-RGC NCδ-Ca^2+^ signal transduction system is functional *in vivo* a mouse model with a disrupted NCδ gene was constructed and analyzed. Unfortunately, disruption of both copies of NCδ gene is lethal. Although it was unexpected at the time of developing the mouse line, it can be now understood in view of the results demonstrating that NCδ may be involved in spermatogenesis. Therefore, heterozygous, NCδ^+/-^, line is viable.

Because earlier studies from our laboratory and others’ had shown that NCδ is expressed in the adrenocortical zona glomerulosa (Nakano et al., 1993; Duda et al., 2012a) and that the adrenal gland also contains functional ANF-RGC transduction system (Takayanagi et al., 1987; Sharma et al., 1989; Duda et al., 1991; Rondeau et al., 1995) the adrenal gland appeared to be the tissue of choice for testing whether the ANF-RGC NCδ-Ca^2+^ signal transduction system is operational and of physiological significance there.

To show that NCδ is indeed the Ca^2+^-sensor modulator of ANF-RGC in the adrenal gland the particulate fractions of the adrenal glands from wild type and NCδ^+/-^ mice and their isogenic controls (NCδ^+/+^) were tested for guanylate cyclase activity in the presence and absence of Ca^2+^. The activity in membranes isolated in the absence of Ca^2+^ was about 66 pmol cyclic GMP min^-1^ (mg protein) ^-1^ for the control and NCδ^+/-^ mice and the activity was not affected by the presence or absence of Ca^2+^ in the assay mixture. However, the activity in membranes isolated in the presence of Ca^2+^ was strongly dependent on the mice genotype and Ca^2+^ in the assay mixture. When assessed in the absence of Ca^2+^, the activity was ~70 pmol cyclic GMP min^-1^ (mg protein) ^-1^ for the weight and NCδ^+/-^ mice but when assessed in the presence of 1 μM Ca^2+^ the activity was 223 ± 20 pmol cyclic GMP min^-1^ (mg protein) ^-1^ for the wild type mice and 135 ± 10 pmol cyclic GMP min^-1^ (mg protein) ^-1^ for the NCδ^+/-^ mice. Thus, the Ca^2+^-dependent NCδ-modulated ANF-RGC signaling pathway in the mice with one copy of NCδ gene deleted (NCδ^+/-^) is functionally half as active as in the wild type mice. To further authenticate that the lowering of the Ca^2+^-dependent cyclase activity in the adrenal gland membranes of NCδ^+/-^ mice is the exclusive consequence of lower NCδ expression, 2 μM exogenous NCδ was added to the NCδ^+/+^ and NCδ^+/-^ membranes (isolated in the presence of Ca^2+^) and the cyclase activity was determined in the presence of 1 μM Ca^2+^. The cyclase activity in the NCδ^+/+^ adrenal membranes increased only minimally, from 220 to 279 ± 21 pmol cyclic GMP min^-1^ (mg protein) ^-1^ but in the NCδ^+/-^ membranes, the increase was significantly larger, from 133 to 284 ± 24 (Duda et al., 2012b). Thus, the activity achieved was practically the same for both types of membranes. Hence, addition of exogenous NCδ to the NCδ^+/-^ adrenal gland membranes restores the guanylate cyclase activity and brings it to the level of activity in the NCδ^+/+^ membranes. We rationalized that the slight activity increase in the NCδ^+/+^ membranes observed upon addition of exogenous NCδ is caused by a partial loss of the native NCδ during the membrane preparation.

What is the function of this pathway in the adrenal gland? In general, the primary role of ANF-RGC in the adrenal gland is to offset the renin-angiotensin system and inhibit aldosterone synthesis, and by doing this, to lower blood pressure (Burnett et al., 1984; Aoki et al., 2000; Shi et al., 2001). Therefore, is the Ca^2+^-dependent ANF-RGC signal transduction machinery in the adrenal gland involved in aldosterone synthesis? Our ongoing studies indicate that indeed it is. The plasma aldosterone levels in the NCδ^+/-^ mice are approximately 27% higher than in the plasma of the control (NCδ^+/+^) mice. And the effect is exclusive for the aldosterone synthesizing glomerulosa cells, because corticosterone (synthesized in fasciculate cells) levels are unaffected by the absence of NCδ gene.

The increased plasma aldosterone level in the NCδ^+/-^ mice correlates with the increase in blood pressure. Systolic blood pressure (measured by the non-invasive tail cuff method) was determined to be 92 ± 6 mm Hg for the wild type mice and 127 ± 9 mm Hg for the NCδ^+/-^ mice. Therefore, the conclusion of these studies was that the ANF-RGC-Ca^2+^-NCδ transduction system is not only physically present but is of physiological significance at least in the adrenal gland.

In summary, this review has high-lighted studies which define the molecular and physiological mechanisms of the hormonal signal transduction of ANF-RGC. In addition, a new signal transduction mechanism and its present state of validation has been narrated. In this model Ca^2+^ is the additional signal of ANF-RGC. By entirely different mechanism, it regulates ANF-RGC catalytic activity and controls its physiological functions.

## FUTURE DIRECTIONS

There are three venues through which, in these authors’ understanding, the future research will progress. The first venue is centered on the basic research to decipher (1) how the ^669^WTAPELL^675^ motif “communicates” with the catalytic domain of ANF-RGC and signals its activation and (2) the mechanism of NCδ-Ca^2+^ signaling of ANF-RGC activity. The second venue relates to the physiology of both ANF-RGC signaling pathways (1) establishing whether the Ca^2+^ signaling mechanism is operative in other tissues in addition to the adrenal gland and (2) to determine the interrelationship of the hormonal and Ca^2+^ pathways. Finally, after deciphering the molecular and physiological details of the two signaling pathway the third venue will be translational, to design a molecule that can target directly the catalytic domain and bring ANF-RGC to its full activity and prevent the pandemic of hypertension, myocardial hypertrophy and obesity. The last objective is the most far-reaching, but hopefully can be achieved after the first two goals are accomplished.

## Conflict of Interest Statement

The authors declare that the research was conducted in the absence of any commercial or financial relationships that could be construed as a potential conflict of interest.

## ABBREVIATIONS

ANF: atrial natriuretic factor
ANF-RGC: atrial natriuretic factor receptor guanylate cyclase
ARM: ATP-regulatory module
ATP: adenosine triphosphate
BNP: B-type natriuretic peptide
CNP: C-type natriuretic peptide
CNP-RGC: C-type natriuretic peptide receptor guanylate cyclase
cyclic AMP: 3′,5′-cyclic adenosine monophosphate
cyclic GMP: 3′,5′-cyclic guanosine monophosphate
CNG channel: cyclic nucleotide gated channel
GCAP: guanylate cyclase activating protein
GC-A: guanylate cyclase-A
GC-B: guanylate cyclase-B
GC-C: guanylate cyclase-C
GC-D: guanylate cyclase-D
GC-E: guanylate cyclase-E
GC-F: guanylate cyclase-F
GC-G: guanylate cyclase-G
KHD: kinase homology domain
K-LD: kinase-like domain
NCδ: neurocalcin δ
NPR-A: natriuretic peptide receptor-A
ONE-GC: olfactory neuroepithelial guanylate cyclase
STa-RGC: heat-stable enterotoxin (also guanylin and uroguanylin) receptor guanylate cyclase
ROS-GC: rod outer segment guanylate cyclase

## References

[B1] Aoki H. Richmond M. Izumo S. Sadoshima J. (2000). Specific role of the extracellular signal-regulated kinase pathway in angiotensin II-induced cardiac hypertrophy in vitro. *Biochem. J.* 347 275–284 10.1042/0264-6021:3470275 10727428 PMC1220957

[B2] Bratová I. Otyepka M. KŘiž Z. KoŘa J. (2005). The mechanism of inhibition of the cyclin-dependent kinase-2 as revealed by the molecular dynamics study on the complex CDK2 with the peptide substrate HHASPRK. *Protein Sci.* 14 445–451 10.1110/ps.04959705 15632290 PMC2253414

[B3] Brenner B. M. Ballermann B. J. Gunning M. E. Zeidel M. L. (1990). Diverse biological actions of atrial natriuretic peptide. *Physiol. Rev.* 7 665–669 2141944 10.1152/physrev.1990.70.3.665

[B4] Burczynska B. Duda T. Sharma R. K. (2007). ATP signaling site in the ARM domain of atrial natriuretic factor receptor guanylate cyclase. *Mol. Cell. Biochem.* 301 193–207 10.1007/s11010-006-9400-7 17277921

[B5] Burnett J. C. Jr., Granger J. P. Opgenorth T. J. (1984). Effect of synthetic atrial natriuretic factor on renal function and rennin release. *Am. J. Physiol.* 247 F863–F866 6238539 10.1152/ajprenal.1984.247.5.F863

[B6] Chang C. H. Kohse K. P. Chang B. Hirata M. Jiang B. Douglas J. E. (1990). Characterization of ATP-stimulated guanylate cyclase activation in rat lung membranes. *Biochim. Biophys. Acta* 1052 159–165 10.1016/0167-4889(90)90071-K 1969749

[B7] Chang M. S. Lowe D. G. Lewis M. Hellmiss R. Chen E. Goeddel D. V. (1989). Differential activation by atrial and brain natriuretic peptides of two different receptor guanylate cyclases. *Nature* 341 68–72 10.1038/341068a0 2570358

[B8] Chao Y.-C. Cheng C.-J. Hsieh H.-T. Lin C.-C. Chen C.-C. Yang R.-B. (2010). Guanylate cyclase-G, expressed in the Grueneberg ganglion olfactory subsystem, is activated by bicarbonate. *Biochem. J.* 432 267–273 10.1042/BJ20100617 20738256

[B9] Chinkers M. Garbers D. L. (1989). The protein kinase domain of the ANP receptor is required for signaling. *Science* 245 1392–1394 10.1126/science.2571188 2571188

[B10] Chinkers M. Garbers D. L. Chang M. S. Lowe D. G. Chin H. M. Goeddel D. V. (1989). A membrane form of guanylate cyclase is an atrial natriuretic peptide receptor. *Nature* 338 78–83 10.1038/338078a0 2563900

[B11] Chinkers M. Singh S. Garbers D. L. (1991). Adenine nucleotides are required for activation of rat atrial natriuretic peptide receptor/guanylyl cyclase expressed in a baculovirus system. *J. Biol. Chem.* 266 4088–4093 1671858

[B12] Cole F. E. Rondon I. Iwata T. Hardee E. Frohlich E. D. (1989). Effect of ATP and amiloride on ANF binding and stimulation of cyclic GMP accumulation in rat glomerular membranes. *Life Sci.* 45 477–484 10.1016/0024-3205(89)90097-0 2570337

[B13] Coussen F. Mulle C. (2006). Kainate receptor-interacting proteins and membrane trafficking. *Biochem. Soc. Trans.* 34 927–930 10.1042/BST0340927 17052229

[B14] Das S. Au E. Krazit S. T. Pandey K. N. (2010). Targeted disruption of guanylyl cyclase-A/natriuretic peptide receptor-A gene provokes renal fibrosis and remodeling in null mutant mice: role of proinflammatory cytokines. *Endocrinology* 151 5841–5850 10.1210/en.2010-0655 20881240 PMC2999494

[B15] de Bold A. J. (1985). Atrial natriuretic factor: a hormone produced by the heart. *Science* 230 767–770 10.1126/science.2932797 2932797

[B16] de Sauvage F. J. Camerato T. R. Goeddel D. V. (1991). Primary structure and functional expression of the human receptor for *Escherichia coli* heat-stable enterotoxin. *J. Biol. Chem.* 266 17912–17918 1680854

[B17] Detwiler P. (2000). Open the loop: dissecting feedback regulation of a second messenger transduction cascade. *Neuron* 36 3–4 10.1016/S0896-6273(02)00940-6 12367499

[B18] Dizhoor A. M. Olshevskaya E. V. Henzel W. J. Wong S. C. Stults J. T. Ankoudinova I. (1995). Cloning, sequencing, and expression of a 24-kDa Ca2+-binding protein activating photoreceptor guanylyl cyclase. *J. Biol. Chem.* 270 25200–25206 10.1074/jbc.270.42.25200 7559656

[B19] Dubois S. K. Kishimoto I. Lillis T. O. Garbers D. L. (2000). A genetic model defines the importance of the atrial natriuretic peptide receptor (guanylyl cyclase-A) in the regulation of kidney function. *Proc. Natl. Acad. Sci. U.S.A.* 97 4369–4373 10.1073/pnas.97.8.4369 10760303 PMC18248

[B20] Duda T. Bharill S. Wojtas I. Yadav P. Gryczynski I. Gryczynski Z. (2009). Atrial natriuretic factor receptor guanylate cyclase signaling: new ATP-regulated transduction motif. *Mol. Cell. Biochem.* 324 39–53 10.1007/s11010-008-9983-2 19137266 PMC2849744

[B21] Duda T. Fik-Rymarkiewicz E. Venkataraman V. Krishnan A. Sharma R. K. (2004). Calcium-modulated ciliary membrane guanylate cyclase transduction machinery: constitution and operational principles. *Mol. Cell. Biochem.* 267 107–122 10.1023/B:MCBI.0000049372.33965.4f 15663192

[B22] Duda T. Goraczniak R. M. Sharma R. K. (1991). Site-directed mutational analysis of a membrane guanylate cyclase cDNA reveals the atrial natriuretic factor signaling site. *Proc. Natl. Acad. Sci. U.S.A.* 88 7882–7886 10.1073/pnas.88.17.7882 1679239 PMC52408

[B23] Duda T. Goraczniak R. M. Sharma R. K. (1993a). The glycine residue of ATP regulatory module in receptor guanylate cyclases that is essential in natriuretic factor signaling. *FEBS Lett.* 335 309–314 10.1016/0014-5793(93)80408-M 7903250

[B24] Duda T. Goraczniak R. M. Sitaramayya A. Sharma R. K. (1993b). Cloning and expression of an ATP-regulated human retina C-type natriuretic factor receptor guanylate cyclase. *Biochemistry* 32 1391–1395 10.1021/bi00057a001 7679284

[B25] Duda T. Goraczniak R. M. Sharma R. K. (1996a). Molecular characterization of S100A1-S100B protein in retina and its activation mechanism of bovine photoreceptor guanylate cyclase. *Biochemistry* 35 6263–6266 10.1021/bi960007m 8639567

[B26] Duda T. Goraczniak R. Surgucheva I. Rudnicka-Nawrot M. Gorczyca W. A. Palczewski K. (1996b). Calcium modulation of bovine photoreceptor guanylate cyclase. *Biochemistry* 35 8478–8482 10.1021/bi960752z 8679607

[B27] Duda T. Jankowska A. Venkataraman V. Nagele R. G. Sharma R. K. (2001a). A novel calcium-regulated membrane guanylate cyclase transduction system in the olfactory neuroepithelium. *Biochemistry* 40 12067–12077 10.1021/bi0108406 11580282

[B28] Duda T. Yadav P. Jankowska A. Venkataraman V. Sharma R. K. (2001b). Three dimensional atomic model and experimental validation for the ATP-Regulated Module (ARM) of the atrial natriuretic factor receptor guanylate cyclase. *Mol. Cell. Biochem.* 217 165–172 10.1023/A:1007236917061 11269661

[B29] Duda T. Krishnan R. Sharma R. K. (2006). GCAP1: antithetical calcium sensor of ROS-GC transduction machinery. *Calcium Bind. Proteins* 1 102–107

[B30] Duda T. Pertzev A. Koch K. W. Sharma R. K. (2012a). Antithetical modes of and the Ca2+ sensors targeting in ANF-RGC and ROS-GC1 membrane guanylate cyclases. *Front. Mol. Neurosci. * 5:44. 10.3389/fnmol.2012.00044 PMC3321476 22509151

[B31] Duda T. Pertzev A. Sharma R. K. (2012b). Ca(2+) modulation of ANF-RGC: new signaling paradigm interlocked with blood pressure regulation. *Biochemistry* 51 9394–9405 10.1021/bi301176c 23088492 PMC3519363

[B32] Duda T. Pertzev A. Sharma R. K. (2013). The ANF-RGC gene motif (669)WTAPELL(675) is vital for blood pressure regulation: Biochemical mechanism. *Biochemistry* 52 2337–2334 10.1021/bi400175d 23464624 PMC3636714

[B33] Duda T. Sharma R. K. (1995). ATP bimodal switch that regulates the ligand binding and signal transduction activities of the atrial natriuretic factor receptor guanylate cyclase. *Biochem. Biophys. Res. Commun.* 209 286–292 10.1006/bbrc.1995.1501 7726848

[B34] Duda T. Sharma R. K. (2005). Two membrane juxtaposed signaling modules in ANF-RGC are interlocked. *Biochem. Biophys. Res. Commun.* 332 149–156 10.1016/j.bbrc.2005.04.102 15896311

[B35] Duda T. Sharma R. K. (2008). ONE-GC membrane guanylate cyclase, a trimodal odorant signal transducer. *Biochem. Biophys. Res. Commun.* 367 440–444 10.1016/j.bbrc.2007.12.153 18178149

[B36] Duda. T. Sharma R. K. (2009). Ca2+-modulated ONE-GC odorant signal transduction. *FEBS Lett.* 583 1327–1330 10.1016/j.febslet.2009.03.036 19306880

[B37] Duda T. Venkataraman V. Ravichandran S. Sharma R. K. (2005). ATP-regulated module (ARM) of the atrial natriuretic factor receptor guanylate cyclase. *Peptides* 26 969–984 10.1016/j.peptides.2004.08.032 15911066

[B38] Duda T. Yadav P. Sharma R. K. (2011). Allosteric modification, the primary ATP activation mechanism of atrial natriuretic factor receptor guanylate cyclase. *Biochemistry* 50 1213–1212 10.1021/bi1018978 21222471 PMC3049299

[B39] Ellis K. L. Newton-Cheh C. Wang T. J. Frampton C. M. Doughty R. N. Whalley G. A. (2011). Association of genetic variation in the natriuretic peptide system with cardiovascular outcomes. *J. Mol. Cell. Cardiol.* 50 695–701 10.1016/j.yjmcc.2011.01.010 21276798

[B40] Ellmers L. J. Scott N. J. Piuhola J. Maeda N. Smithies O. Frampton C. M. (2007). Npr1-regulated gene pathways contributing to cardiac hypertrophy and fibrosis. *J. Mol. Endocrinol.* 38 245–257 10.1677/jme.1.02138 17293444

[B41] Foster D. C. Garbers D. L. (1998). Dual role for adenine nucleotides in the regulation of the atrial natriuretic peptide receptor, guanylyl cyclase-A. *J. Biol. Chem.* 273 16311–16318 10.1074/jbc.273.26.16311 9632692

[B42] Fulle H. J. Vassar R. Foster D. C. Yang R. B. Axel R. Garbers D. L. (1995). A receptor guanylyl cyclase expressed specifically in olfactory sensory neurons. *Proc. Natl. Acad. Sci. U.S.A.* 92 3571–3575 10.1073/pnas.92.8.3571 7724600 PMC42209

[B43] Goraczniak R. M. Duda T. Sharma R. K. (1992). A structural motif that defines the ATP-regulatory module of guanylate cyclase in atrial natriuretic factor signalling. *Biochem. J.* 282 533–537 1347681 10.1042/bj2820533 PMC1130813

[B44] Goraczniak R. M. Duda T. Sharma R. K. (1998). Calcium modulated signaling site in type 2 rod outer segment membrane guanylate cyclase (ROS-GC2). *Biochem. Biophys. Res. Commun.* 245 447–453 10.1006/bbrc.1998.8455 9571173

[B45] Goraczniak R. M. Duda T. Sitaramayya A. Sharma R. K. (1994). Structural and functional characterization of the rod outer segment membrane guanylate cyclase. *Biochem. J.* 302 455–461 7916565 10.1042/bj3020455 PMC1137250

[B46] Hamra F. K. Forte L. R. Eber S. L. Pidhorodeckyj N. V. Krause W. J. Freeman R. H. (1993). Uroguanylin: structure and activity of a second endogenous peptide that stimulates intestinal guanylate cyclase. *Proc. Natl. Acad. Sci. U.S.A.* 90 10464–10468 10.1073/pnas.90.22.10464 7902563 PMC47797

[B47] Hanks S. K. Quinn A. M. Hunter T. (1988). The protein kinase family: conserved features and deduced phylogeny of the catalytic domains. *Science* 241 42–52 10.1126/science.3291115 3291115

[B48] He X. Nishio K. Misono K. S. (1995). High-yield affinity alkylation of the atrial natriuretic factor receptor binding site. *Bioconjug. Chem.* 6 541–548 10.1021/bc00035a007 8974452

[B49] Holtwick R. van Eickels M. Skryabin B. V. Baba H. A. Bubikat A. Begrow F. (2003). Pressure independent cardiac hypertrophy in mice with cardiomyocyte-restricted inactivation of the atrial natriuretic peptide receptor guanyly cyclase-A. *J. Clin. Invest.* 111 1399–1407 10.1172/JCI17061 12727932 PMC154444

[B50] Huo X. Abe T. Misono K. S. (1999). Ligand binding-dependent limited proteolysis of the atrial natriuretic peptide receptor: juxtamembrane hinge structure essential for transmembrane signal transduction. *Biochemistry* 38 16941–16951 10.1021/bi9919448 10606529

[B51] Iino S. Kobayashi S. Okazaki K. Hidaka H. (1995). Immunohistochemical localization of neurocalcin in the rat inner ear. *Brain Res.* 680 128–134 10.1016/0006-8993(95)00253-M 7663968

[B52] Ivings L. Pennington S. R. Jenkins R. Weiss J. L. Burgoyne R. D. (2002). Identification of Ca2+-dependent binding partners for the neuronal calcium sensor protein neurocalcin delta: interaction with actin, clathrin and tubulin. *Biochem. J.* 363 599–608 10.1042/0264-6021:3630599 11964161 PMC1222513

[B53] Katholi R. E. Couri D. M. (2011). Left ventricular hypertrophy: major risk factor in patients with hypertension: update and practical clinical applications. *Int. J. Hypertens.* 2011 495349 10.4061/2011/495349 PMC3132610 21755036

[B54] Khare S. Wilson D. Wali R. K. Tien X. Y. Bissonnette M. Niedziela S. M. (1994). Guanylin activates rat colonic particulate guanylate cyclase. *Biochem. Biophys. Res. Commun.* 203 1432–1437 10.1006/bbrc.1994.2345 7945291

[B55] Kishimoto I. Rossi K. Garbers D. L. (2001). A genetic model provides evidence that the receptor for atrial natriuretic peptide (guanylyl cyclase-A) inhibits cardiac ventricular myocyte hypertrophy. *Proc. Natl. Acad. Sci. U.S.A.* 98 2703–2706 10.1073/pnas.051625598 11226303 PMC30202

[B56] Knowles J. W. Esposito G. Mao L. Hagaman J. R. Fox J. E. Smithies O. (2001). Pressure independent enhancement of cardiac hypertrophy in natriuretic peptide receptor A-deficient mice. *J. Clin. Invest.* 107 975–984 10.1172/JCI11273 11306601 PMC199554

[B57] Koch K.-W. (1991). Purification and identification of photoreceptor guanylate cyclase. *J. Biol. Chem.* 266 8634–8637 1673683

[B58] Koch K.-W. Duda T. Sharma R. K. (2010). Ca2+-modulated vision-linked ROS-GC guanylate cyclase transduction machinery. *Mol. Cell. Biochem.* 334 105–115 10.1007/s11010-009-0330-z 19943184

[B59] Kuhn M. Ng C. K. Su Y. H. Kilić A. Mitko D. Bien-Ly N. (2004). Identification of an orphan guanylate cyclase receptor selectively expressed in mouse testis. *Biochem. J.* 379 385–393 10.1042/BJ20031624 14713286 PMC1224077

[B60] Kumar V. D. Vijay-Kumar S. Krishnan A. Duda T. Sharma R. K. (1999). A second calcium regulator of rod outer segment membrane guanylate cyclase, ROS-GC1: neurocalcin. *Biochemistry* 38 12614–12620 10.1021/bi990851n 10504230

[B61] Krishnan A. Venkataraman V. Fik-Rymarkiewicz E. Duda T. Sharma R. K. (2004). Structural, biochemical, and functional characterization of the calcium sensor neurocalcin delta in the inner retinal neurons and its linkage with the rod outer segment membrane guanylate cyclase transduction system. *Biochemistry* 43 2708–2723 10.1021/bi035631v 15005606

[B62] Kuno T. Andresen J. W. Kamisaki Y. Waldman S. A. Chang L. Y. Saheki S. (1986). Co-purification of an atrial natriuretic factor receptor and particulate guanylate cyclase from rat lung. *J. Biol. Chem.* 261 5817–5823 2871018

[B63] Kurose H. Inagami T. Ui M. (1987). Participation of adenosine 5^′^-triphosphate in the activation of membrane-bound guanylate cyclase by the atrial natriuretic factor. *FEBS Lett.* 219 375–379 10.1016/0014-5793(87)80256-9 2886366

[B64] Labrecque J. Deschênes J. McNicoll N De Léan A. (2001). Agonistic induction of a covalent dimer in a mutant of natriuretic peptide receptor-A documents a juxtamembrane interaction that accompanies receptor activation. *J. Biol. Chem.* 276 8064–8072 10.1074/jbc.M005550200 11124937

[B65] Labrecque J. Mc Nicoll N. Marquis M De Lean A. (1999). A disulfide-bridged mutant of natriuretic peptide receptor-A displays constitutive activity. Role of receptor dimerization in signal transduction.* J. Biol. Chem.* 274 9752–9759 10.1074/jbc.274.14.9752 10092664

[B66] Ladant D. (1995). Calcium and membrane binding properties of bovine neurocalcin delta expressed in *Escherichia coli*. *J. Biol. Chem.* 270 3179–3185 7852401

[B67] Larose L. McNicoll N. Ong H De Léan A. (1991). Allosteric modulation by ATP of the bovine adrenal natriuretic factor R1 receptor functions. *Biochemistry* 30 8990–8995 10.1021/bi00101a012 1654083

[B68] Leinders-Zufall T. Cockerham R. E. Michalakis S. Biel M. Garbers D. L. Reed R. R. (2007). Contribution of the receptor guanylyl cyclase GC-D to chemosensory function in the olfactory epithelium. *Proc. Natl. Acad. Sci. U.S.A.* 104 14507–14512 10.1073/pnas.0704965104 17724338 PMC1964822

[B69] Liu Y. Ruoho A. E. Rao V. D. Hurley J. H. (1997). Catalytic mechanism of the adenylyl and guanylyl cyclases: modeling and mutational analysis. *Proc. Natl. Acad. Sci. U.S.A.* 94 13414–13419 10.1073/pnas.94.25.13414 9391039 PMC28319

[B70] Lowe D. G. Chang M. S. Hellmiss R. Chen E. Singh S. Garbers D. L. (1989). Human atrial natriuretic peptide receptor defines a new paradigm for second messenger signal transduction. *EMBO J.* 8 1377–13784 2569967 10.1002/j.1460-2075.1989.tb03518.x PMC400964

[B71] Lowe D. G. Dizhoor A. M. Liu K. Gu Q. Spencer M. Laura R. (1995). Cloning and expression of a second photoreceptor-specific membrane retina guanylyl cyclase (RetGC), RetGC-2. *Proc. Natl. Acad. Sci. U.S.A.* 92 5535–5539 10.1073/pnas.92.12.5535 7777544 PMC41730

[B72] Maack T. (1996). Role of atrial natriuretic factor in volume control. *Kidney Int.* 49 1732–1737 10.1038/ki.1996.257 8743487

[B73] Mamasuew K. Breer H. Fleischer J. (2008). Gruenberg ganglion neurons respond to cool ambient temperatures. *Eur. J. Neurosci.* 28 1775–1785 10.1111/j.1460-9568.2008.06465.x 18973593

[B74] Marala R. Duda T. Goraczniak R. M. Sharma R. K. (1992). Genetically tailored atrial natriuretic factor-dependent guanylate cyclase. Immunological and functional identity with 180 kDa membrane guanylate cyclase and ATP signaling site. *FEBS Lett.* 296 254–258 10.1016/0014-5793(92)80298-U 1347019

[B75] Marala R. B. Sitaramayya A. Sharma R. K. (1991). Dual regulation of atrial natriuretic factor-dependent guanylate cyclase activity by ATP. *FEBS Lett.* 281 73–76 10.1016/0014-5793(91)80361-6 1673103

[B76] Margulis A. Pozdnyakov N. Sitaramayya A. (1996). Activation of bovine photoreceptor guanylate cyclase by S100 proteins. *Biochem. Biophys. Res. Commun.* 218 243–247 10.1006/bbrc.1996.0043 8573140

[B77] McNicoll N. Escher E. Wilkes B. C. Schiller P. W. Ong H De Léan A. (1992). Highly efficient photoaffinity labeling of the hormone binding domain of atrial natriuretic factor receptor. *Biochemistry* 31 4487–4493 10.1021/bi00133a015 1316147

[B78] McNicoll N. Gagnon J. Rondeau J. J. Ong H De Léan A. (1996). Localization by photoaffinity labeling of natriuretic peptide receptor-A binding domain. *Biochemistry* 35 12950–12956 10.1021/bi960818q 8841141

[B79] Meloche S. McNicoll N. Liu B. Ong H De Lean A. (1988). Atrial natriuretic factor R1 receptor from bovine adrenal zona glomerulosa: purification, characterization, and modulation by amiloride. *Biochemistry* 27 8151–8158 10.1021/bi00421a025 2852953

[B80] Miao Z. H. Song D. L. Douglas J. G. Chang C. H. (1995). Mutational inactivation of the catalytic domain of guanylate cyclase-A receptor. *Hypertension* 25 694–698 10.1161/01.HYP.25.4.694 7721418

[B81] Misono K. S. (2002). Natriuretic peptide receptor: structure and signaling. *Mol. Cell. Biochem.* 230 49–60 10.1023/A:1014257621362 11952096

[B82] Misono K. S. Ogawa H. Qiu Y. Ogata C. M. (2005). Structural studies of the natriuretic peptide receptor: a novel hormone-induced rotation mechanism for transmembrane signal transduction. *Peptides* 26 957–968 10.1016/j.peptides.2004.12.021 15911065

[B83] Misono K. S. Philo J. S. Arakawa T. Ogata C. M. Qiu Y. Ogawa H. (2011). Structure, signaling mechanism and regulation of the natriuretic peptide receptor guanylate cyclase. *FEBS J.* 278 1818–1829 10.1111/j.1742-4658.2011.08083.x 21375693 PMC3097287

[B84] Miyagi M. Misono K. S. (2000). Disulfide bond structure of the atrial natriuretic peptide receptor extracellular domain: conserved disulfide bonds among guanylate cyclase-coupled receptors. *Biochim. Biophys. Acta* 1478 30–38 10.1016/S0167-4838(00)00002-9 10719172

[B85] Nakano A. Terasawa M. Watanabe M. Okazaki K. Inoue S. Kato M. (1993). Distinct regional localization of neurocalcin, a Ca2+-binding protein, in the bovine adrenal gland. *J. Endocrinol.* 138 283–290 10.1677/joe.0.1380283 8228737

[B86] Ogawa H. Qiu Y. Huang L. Tam-Chang S. W. Young H. S. Misono K. S. (2009). Structure of the atrial natriuretic peptide receptor extracellular domain in the unbound and hormone-bound states by single-particle electron microscopy. *FEBS J.* 276 1347–1355 10.1111/j.1742-4658.2009.06870.x 19187227

[B87] Ogawa H. Qiu Y. Ogata C. M. Misono K. S. (2004). Crystal structure of hormone-bound atrial natriuretic peptide receptor extracellular domain: rotation mechanism for transmembrane signal transduction. *J. Biol. Chem.* 279 28625–28631 10.1074/jbc.M313222200 15117952

[B88] Ogawa H. Zhang X. Qiu Y. Ogata C. M. Misono K. S. (2003). Crystallization and preliminary X-ray analysis of the atrial natriuretic peptide (ANP) receptor extracellular domain complex with ANP: use of ammonium sulfate as the cryosalt. *Acta Crystallogr. D Biol. Crystallogr.* 59 1831–1833 10.1107/S0907444903016445 14501129

[B89] Okazaki K. Obata N. H. Inoue S. Hidaka H. (1995). S100 beta is a target protein of neurocalcin delta, an abundant isoform in glial cells. *Biochem. J.* 306 551–555 7887910 10.1042/bj3060551 PMC1136553

[B90] Oliver P. M. Fox J. E. Kim R. Rockman H. A. Kim H. S. Reddick R. L. (1997). Hypertension, cardiac hypertrophy, and sudden death in mice lacking natriuretic peptide receptor A. *Proc. Natl. Acad. Sci. U.S.A.* 94 14730–14735 10.1073/pnas.94.26.14730 9405681 PMC25105

[B91] Olson L. J. Ho B. Cashdollar L. W. Drewett J. G. (1998). Functionally active catalytic domain is essential of guanylyl cyclase-linked receptor mediated inhibition of human aldosterone synthesis. *Mol. Pharmacol.* 54 761–769 9804611 10.1124/mol.54.5.761

[B92] Palczewski K. Subbaraya I. Gorczyca W. A. Helekar B. S. Ruiz C. C. Ohguro H. (1994). Molecular cloning and characterization of retinal photoreceptor guanylyl cyclase-activating protein. *Neuron* 13 395–404 10.1016/0896-6273(94)90355-7 7520254

[B93] Pandey K. N. (2011). Guanylyl cyclase/atrial natriuretic peptide receptor-A: role in the pathophysiology of cardiovascular regulation. *Can. J. Physiol. Pharmacol.* 89 557–573 10.1139/y11-054 21815745 PMC3345283

[B94] Pandey K. N. Singh S. (1990). Molecular cloning and expression of murine guanylate cyclase/atrial natriuretic factor receptor cDNA. *J. Biol. Chem.* 265 12342–12348 1973687

[B95] Parat M. Blanchet J De Léan A. (2010). Role of juxtamembrane and transmembrane domains in the mechanism of natriuretic peptide receptor A activation. *Biochemistry* 49 4601–4610 10.1021/bi901711w 20214400

[B96] Paul A. K. (1986). *Particulate Guanylate Cyclase from Adrenocortical Carcinoma 494. Purification, Biochemical and Immunological Characterization*. Ph.D. thesis, University of Tennessee

[B97] Paul A. K. Marala R. B. Jaiswal R. K. Sharma R. K. (1987). Coexistence of guanylate cyclase and atrial natriuretic factor receptor in a 180-kD protein. *Science* 235 1224–1226 10.1126/science.2881352 2881352

[B98] Pertzev A. Duda T. Sharma R. K. (2010). Ca2+ sensor GCAP1: a constitutive element of the ONE-GC-modulated odorant signal transduction pathway. *Biochemistry* 49 7303–7313 10.1021/bi101001v 20684533 PMC2936275

[B99] Potter L. R. Hunter T. (1998a). Phosphorylation of the kinase homology domain is essential for activation of the A-type natriuretic peptide receptor. *Mol. Cell. Biol.* 18 2164–2172 9528788 10.1128/mcb.18.4.2164 PMC121455

[B100] Potter L. R. Hunter T. (1998b). Identification and characterization of the major phosphorylation sites of the B-type natriuretic peptide receptor. *J. Biol. Chem.* 273 15533–15539 10.1074/jbc.273.25.15533 9624142

[B101] Potter L. R. Hunter T. (1999a). Identification and characterization of the phosphorylation sites of the guanylyl cyclase-linked natriuretic peptide receptors A and B. *Methods* 19 506–520 10.1006/meth.1999.0893 10581150

[B102] Potter L. R. Hunter T. (1999b). A constitutively ``phosphorylated'' guanylyl cyclase-linked atrial natriuretic peptide receptor mutant is resistant to desensitization. *Mol. Biol. Cell* 10 1811–1820 10.1091/mbc.10.6.1811 10359598 PMC25375

[B103] Pozdnyakov N. Yoshida A. Cooper N. G. Margulis A. Duda T. Sharma R. K. (1995). A novel calcium-dependent activator of retinal rod outer segment membrane guanylate cyclase. *Biochemistry* 34 14279–14283 10.1021/bi00044a002 7578029

[B104] Pugh E. N. Jr., Duda T. Sitaramayya A. Sharma R. K. (1997). Photoreceptor guanylate cyclases: a review. *Biosci. Rep.* 17 429–473 10.1023/A:1027365520442 9419388

[B105] Rebello M. R. Aktas A. Medler K. F. (2011). Expression of calcium binding proteins in mouse type II taste cells. *J. Histochem. Cytochem.* 59 530–539 10.1369/0022155411402352 21527586 PMC3201173

[B106] Rondeau J. J. McNicoll N. Gagnon J. Bouchard N. Ong H De Léan A. (1995). Stoichiometry of the atrial natriuretic factor-R1 receptor complex in the bovine zona glomerulosa. *Biochemistry* 34 2130–2136 10.1021/bi00007a005 7857923

[B107] Schulz S. Green C. K. Yuen P. S. Garbers D. L. (1990). Guanylyl cyclase is a heat-stable enterotoxin receptor. *Cell* 63 941–948 10.1016/0092-8674(90)90497-3 1701694

[B108] Schulz S. Singh S. Bellet R. A. Singh G. Tubb D. J. Chin H. (1989). The primary structure of a plasma membrane guanylate cyclase demonstrates diversity within this new receptor family. *Cell* 58 1155–1162 10.1016/0092-8674(89)90513-8 2570641

[B109] Schulz S. Wedel B. J. Matthews A. Garbers D. L. (1998). The cloning and expression of a new guanylyl cyclase orphan receptor. *J. Biol. Chem.* 273 1032–1037 10.1074/jbc.273.2.1032 9422765

[B110] Sharma R. K. (2002). Evolution of the membrane guanylate cyclase transduction system. *Mol. Cell. Biochem.* 230 3–30 10.1023/A:1014280410459 11952094

[B111] Sharma R. K. (2010). Membrane guanylate cyclase is a beautiful signal transduction machine: overview. *Mol. Cell. Biochem.* 334 3–36 10.1007/s11010-009-0336-6 19957201

[B112] Sharma R. K. Duda T. (2010). Odorant-linked ROS-GC subfamily membrane guanylate cyclase transduction system. *Mol. Cell. Biochem.* 334 181–189 10.1007/s11010-009-0333-9 19937091

[B113] Sharma R. K. Jaiswal R. K. Duda T. (1988a). ``Second messenger role of cyclic GMP in atrial natriuretic factor receptor mediated signal transduction: 180 kDa membrane guanylate cyclase, its coupling with atrial natriuretic factor receptor and its regulation by protein kinase C,'' in *Biological and Molecular Aspects of Atrial Factors* ed. Needleman (New york:Alan R. Liss, Inc.) 77–96

[B114] Sharma R. K. Marala R. B. Paul A. K. (1988b). ``Mediatory role of cyclic GMP in receptor-mediated signal transduction: membrane guanylate cyclase and its coupling with atrial natriuretic factor receptor,'' in *American Society of Hypertension Symposium Series, Advances in Peptide Research*, Vol 2 eds Brenner B. M. Laragh J. H. (New York:Raven Press) 61–77

[B115] Sharma R. K. Marala R. B. Duda T. (1989). Purification and characterization of the 180-kDa membrane guanylate cyclase containing atrial natriuretic factor receptor from rat adrenal gland and its regulation by protein kinase C. *Steroids* 53 437–460 10.1016/0039-128X(89)90024-X 2572076

[B116] Sharma R. K. Yadav P. Duda T. (2001). Allosteric regulatory step and configuration of the ATP-binding pocket in atrial natriuretic factor receptor guanylate cyclase transduction mechanism. *Can. J. Physiol. Pharmacol.* 79 682–691 10.1139/y01-033 11558677

[B117] Shi S.-J Nguyen H. T. Sharma G. D. Navar G. Pandey K. N. (2001). Genetic disruption of atrial natriuretic peptide receptor-A alters rennin and angiotensinII levels. *Am. J. Physiol. Renal Physiol.* 281 F665–F673 11553513 10.1152/ajprenal.2001.281.4.F665

[B118] Shyjan A. W. de Sauvage F. J. Gillett N. A. Goeddel D. V. Lowe D. G. (1992). Molecular cloning of a retina-specific membrane guanylyl cyclase. *Neuron* 9 727–737 10.1016/0896-6273(92)90035-C 1356371

[B119] Singh S. Singh G. Heim J. M. Gerzer R. (1991). Isolation and expression of a guanylate cyclase-coupled heat stable enterotoxin receptor cDNA from a human colonic cell line. *Biochem. Biophys. Res. Commun.* 179 1455–1463 10.1016/0006-291X(91)91736-V 1718270

[B120] Takayanagi R. Inagami T. Snajdar R. M. Imada T. Tamura M. Misono K. S. (1987). Two distinct forms of receptors for atrial natriuretic factor in bovine adrenocortical cells. Purification, ligand binding, and peptide mapping.* J. Biol. Chem.* 262 12104–12113 2887565

[B121] Thompson D. K. Garbers D. L. (1995). Dominant negative mutations of the guanylyl cyclase-A receptor. Extracellular domain deletion and catalytic domain point mutations.* J. Biol. Chem.* 270 425–430 10.1074/jbc.270.1.425 7814405

[B122] Thorpe D. S. Morkin E. (1990). The carboxyl region contains the catalytic domain of the membrane form of guanylate cyclase. *J. Biol. Chem.* 265 14717–14720 1975586

[B123] Thorpe D. S. Niu S. Morkin E. (1996). The guanylyl cyclase core of an atrial natriuretic peptide receptor: enzymatic properties and basis for cooperativity. *Biochem. Biophys. Res. Commun.* 218 670–673 10.1006/bbrc.1996.0120 8579572

[B124] Tremblay J. Huot C. Koch C. Potier M. (1991). Characterization of the functional domains of the natriuretic peptide receptor/guanylate cyclase by radiation inactivation. *J. Biol. Chem.* 266 8171–8175 1673679

[B125] Tucker C. L. Hurley J. H. Miller T. R. Hurley J. B. (1998). Two amino acid substitutions convert a guanylyl cyclase, RetGC-1, into an adenylyl cyclase. *Proc. Natl. Acad. Sci. U.S.A.* 95 5993–5997 10.1073/pnas.95.11.5993 9600905 PMC27573

[B126] Venkataraman V. Duda T. Ravichandran S. Sharma R. K. (2008). Neurocalcin delta modulation of ROS-GC1, a new model of Ca2+ signaling. *Biochemistry* 47 6590–6601 10.1021/bi800394s 18500817 PMC2844899

[B127] Vijay-Kumar S. Kumar V. D. (1999). Crystal structure of recombinant bovine neurocalcin. *Nat. Struct. Biol.* 6 80–88 10.1038/4956 9886296

[B128] Wadei H. M. Textor S. C. (2012). The role of the kidney in regulating arterial blood pressure. *Nat. Rev. Nephrol.* 8 602–609 10.1038/nrneph.2012.191 22926246

[B129] Wedel B. J. Foster D. C. Miller D. E. Garbers D. L. (1997). A mutation of the atrial natriuretic peptide (guanylyl cyclase-A) receptor results in a constitutively hyperactive enzyme. *Proc. Natl. Acad. Sci. U.S.A.* 94 459–462 10.1073/pnas.94.2.459 9012805 PMC19534

[B130] Wedel B. J. Garbers D. L. (1997). New insights on the functions of the guanylyl cyclase receptors. *FEBS Lett.* 410 29–33 10.1016/S0014-5793(97)00358-X 9247117

[B131] Wen X. H. Duda T. Pertzev A. Venkataraman V. Makino C. L. Sharma R. K. (2012). S100B serves as a Ca2+ sensor for ROS-GC1 guanylate cyclase in cones but not in rods of the murine retina. *Cell. Physiol. Biochem.* 29 417–430 10.1159/000338496 22508049 PMC3434333

[B132] Wierenga R. K. Hol W. G. (1983). Predicted nucleotide-binding properties of p21 protein and its cancer-associated variant. *Nature* 302 842–844 10.1038/302842a0 6843652

[B133] Wilson E. M. Chinkers M. (1995). Identification of sequences mediating guanylyl cyclase dimerization. *Biochemistry* 34 4696–4701 10.1021/bi00014a025 7718574

[B134] Wong S. K. Ma C. P. Foster D. C. Chen A. Y. Garbers D. L. (1995). The guanylyl cyclase-A receptor transduces an atrial natriuretic peptide/ATP activation signal in the absence of other proteins. *J. Biol. Chem.* 270 30818–30822 10.1074/jbc.270.51.30818 8530525

[B135] Yang R. B. Foster D. C Garbers D. L. Fulle H. J. (1995). Two membrane forms of guanylyl cyclase found in the eye. *Proc. Natl. Acad. Sci. U.S.A.* 92 602–606 10.1073/pnas.92.2.602 7831337 PMC42790

[B136] Zhao D. Vellaichamy E. Somanna N. K. Pandey K. N. (2007). Guanylyl cyclase/natriuretic peptide receptor-A gene disruption causes increased adrenal angiotensin II and aldosterone levels. *Am. J. Physiol. Renal Physiol.* 293 F121–F127 10.1152/ajprenal.00478.2006 17389676

[B137] Zozulya S. Stryer L. (1992). Calcium-myristoyl protein switch. *Proc. Natl. Acad. Sci. U.S.A.* 89 11569–11573 10.1073/pnas.89.23.11569 1454850 PMC50594

[B138] Zufall F. Munger S. D. (2010). Receptor guanylyl cyclases in mammalian olfactory function. *Mol. Cell. Biochem.* 334 191–197 10.1007/s11010-009-0325-9 19941039 PMC2809823

